# Factors affecting students’ entrepreneurial intentions: a systematic review (2005–2022) for future directions in theory and practice

**DOI:** 10.1007/s11301-022-00289-2

**Published:** 2022-08-15

**Authors:** Greeni Maheshwari, Khanh Linh Kha, Anantha Raj A. Arokiasamy

**Affiliations:** 1 Economics and Finance Department, RMIT University, Ho Chi Minh City, Vietnam; 2 School of Economics and Management, Xiamen University, Sepang, Selangor Malaysia

**Keywords:** Entrepreneurial intentions, Systematic literature review, Citation analysis, Thematic analysis, Entrepreneurship, A22, A23, L26, O50

## Abstract

Entrepreneurship has been viewed as a critical contributor and an economic engine in a country for creating new jobs and it is crucial for graduates to alter their mindset to become self-employed. Thus, it is necessary to synthesize the factors that impact the entrepreneurial intentions (EI) of students at tertiary level. The aim of this research is twofold; first to identify the factors which have been most studied in the literature and second, to determine which factors are less explored to measure the EI of students. This research adopts the systematic review approach to identify various studies conducted between 2005 to June 2022. The paper further adopted citation analysis and identified the 36 most impactful studies in this area of research. Next, the thematic analysis was conducted and seven main themes (factors) (cognitive, personality, environmental, social, educational, contextual and demographic) of EI determinants were identified. The analysis of the papers clearly demonstrated that the TPB model and cognitive factors dominate this area of research. Furthermore, over half of the studies are conducted in Asia, hence it is important to explore other regions such as Africa, America and Europe and other comparative studies between various regions. The study offers avenues for future research and practical implications of the study for the practitioners.

## Introduction

Entrepreneurship has been viewed as a critical contributor and an economic engine of every country as it helps in creating new jobs, and increases innovation and competitiveness in the labor market (Barba-Sánchez et al. 2022). Entrepreneurship activities have been given importance in many Western countries and are also gaining more attention in developing countries. Many studies have identified entrepreneurial intention (EI) as one of the most significant predictors of entrepreneurial activities and behaviors (Krueger et al. 2000; Autio et al. 2001; Arasti et al. 2012). Hence, the focus of various contemporary research has shifted from entrepreneurship to EI (Yu et al. 2021). Indeed, the number of studies using EI as a research framework has increased since the early 90s, confirming the importance of EI aspect in several settings (Liñán and Fayolle 2015). In addition, it is crucial for graduates to eventually alter their mindset from searching for jobs to creating jobs as a country’s government will not be able to ensure sufficient job provision for all tertiary-level graduates in the future (Reuel Johnmark et al. 2016). University students should shift their focus towards entrepreneurial revolution (Nuan and Xin 2012; Jiang and Sun 2015). Considering this, it is important to understand the factors that affect the EI of students in order to nurture their future entrepreneurialism in their respective countries.

There have been several studies conducted by scholars to examine the factors that impact EI of higher education students. Cognitive and personality factors, such as self-efficacy, individual attitudes, desire for achievement and behavioral control, have significant influence on students’ intentions towards entrepreneurship (Nasip et al. 2017; Shah and Soomro 2017; Biswas and Verma 2021). Social and environmental researchers have identified elements such as prior experience, family background, regional culture and government support as critical factors that affect EI of students (Ahamed and Rokhman 2015; Ali et al. 2019; Tiwari et al. 2020). Another fundamental factor contributing to the formation of students’ EI is entrepreneurial education. Entrepreneurial education in higher education plays an important role in enhancing foundational entrepreneurial knowledge and various cognitive and non-cognitive skills by stimulating students’ entrepreneurial activities (Walter and Block 2016; Brüne and Lutz 2020). This will further motivate students towards entrepreneurship, help improve entrepreneurship quality, and lead to entrepreneurial success (Galloway and Brown 2002). Many entrepreneurship models and theories have been developed to investigate the impact of factors on EI of an individual. Among those proposed models, most of the papers in this research area used theory of planned behaviour (TPB) model (as highlighted by analysis from this review discussed next in the paper) to study the EI of the students. The EI of students is not only affected by factors from TPB model, but there are various other models affecting the EI of students as discussed later in the paper.

Based on the different models developed on entrepreneurship (as discussed further in the paper), there are various factors that affect the entrepreneurial intentions of university students, such as educational factors, contextual factors, environmental factors, psychological factors, and personality factors but little work is done to understand which factors scholars considered the most in measuring the entrepreneurial intentions of the university students. This review of literature is based on the synthesis of papers and will provide an overview of (1) which factors the scholars have paid the most attention to measure EI of the students and (2) what factors are understudied in the literature to determine the factors affecting EI of students.

This study will contribute to the growing body of literature on the factors affecting entrepreneurial intentions of university students by providing theoretical and practical contributions. The study will be particularly beneficial to researchers working in this research area as this paper also provides gaps for future research. The study results would also help educational institutions to support and encourage students towards their entrepreneurial intentions and policy makers who will be able to understand how they can support the development of entrepreneurship activities that in turn, enhance the economic growth of the country.

Despite entrepreneurship being considered a key contributor for sustained growth and development of countries, and EI being regarded as the dominant influence of an individual’s entrepreneurship, the non-quantitative studies pertaining to determine the factors affecting EI have not been paid much attention. There are some scholars who have conducted systematic reviews of study in this area; for example, Pittaway and Cope (2007) examined the interface between higher education institutions and business sector. The study by Bae et al. (2014) was regarding a systematic review of literature in order to find the correlation between entrepreneurial education and EI of students. Nabi et al. (2017) provided a systematic review of literature to study the impact on the EI of students considering entrepreneurial education. Wu and Wu (2017) systematically reviewed the effect of entrepreneurial education on the EI of students, particularly in the Asia–Pacific region. It is important to synthesize the literature to get the holistic picture and contribute towards this research field (Kuckertz and Block 2021). Hence, in this paper, the synthesis of factors that impact the EI of students is carried out and further analyze the extent they have been used by various scholars in this field.

This research adopts the systematic review approach, which is important for synthesizing knowledge to identify numerous studies conducted between 2005 and June 2022. The studies on the EI of students have been receiving a lot of attention since 2016 (as determined by the analysis in this paper) and hence it is clearly visible that this area triggered the researchers’ interest and therefore is important to understand which factors are considered by different scholars to measure the EI of university students in the studies conducted so far. The aim of this research is twofold; first to identify the factors affecting the EI of students which have been studied the most in literature in previous years (from 2005) across the world. Second, to determine which factors are less explored in measuring the entrepreneurial intentions of students and thus can be explored more in future studies. A clearer perspective regarding various factors affecting EI of university students used by various scholars are analyzed in this paper. The findings from this paper can support practitioners to implement policies and take action to promote entrepreneurship in higher education students, as well as provide insights for further research in the future.

Following this brief introduction, the rest of the paper is organized as follows. Section 2 displays the review of literature on the development of various entrepreneurial models, Sect. 3 explains the research methodology, while Sect. 4 provides the results of the review, the next section corresponds to the discussion of the paper and the concluding remarks are provided in the last section of the paper.

## Review of development of entrepreneurial models

There are various models developed and used by various scholars to determine the entrepreneurial intentions of an individual.

### The entrepreneurial event model (EEM)

The entrepreneurial event model (EEM) proposed by Shapero and Sokol (1982) is the first model to shed light on entrepreneurial intention theory. According to the model, the three main determinants that affect an individual’s intention in entrepreneurship are perceived desirability, perceived feasibility and propensity to act. The perception of desirability towards entrepreneurship implies the engagingness of entrepreneurial conduct that a person can perceive. Perceived feasibility signifies the extent that an individual believes they can perform entrepreneurial behavior; and propensity to act indicates the possibility to become an entrepreneur. The proposed model also highlights that the “entrepreneurial event” acts as a trigger to determine behavior towards entrepreneurship of an individual, which can help them to make the best decision among a range of choices.

### The expectancy theory

Expectancy Theory (known as Theory of Motivation or the Rational Intention Theory) developed by (Vroom (1964) states that conscious choice of an individual to maximize satisfaction and minimize adversity will lead to a person’s behavior. In the theory, motivation is defined as a result of an expectancy that greater effort will foster greater performance, instrumentality refers to the expectation of an individual to receive a certain outcome when they make the effort, and valence implies the degree to which the person values the outcome. The Expectancy Theory has been used as a framework in many studies to explore people’s motivation for becoming entrepreneurs (Locke and Baum 2007). The three variables: expectancy, instrumentality, and valence, were confirmed to increase entrepreneurship motivation, further concluding that apart from ability and aptitude, motivation could enhance the entrepreneurial intentions of an individual (Barba-Sánchez and Atienza-Sahuquillo 2017, 2018).

### The theory of planned behavior model (TPB)

The theory of planned behavior (TPB) is advanced from the theory of reasoned action (TRA) by Ajzen and Fishbein (1980). TRA implies that intentions, which are shaped by personal attitudes and subjective norms, will govern the actions of an individual. Regarding the TPB model by Ajzen (1991), the behavior of a person is based on voluntary control and specific planning. TPB defines the three antecedents that shape an individual's intention, namely attitudes towards behavior (ATB), social norms (SN), and perceived behavioral control (PBC). ATB implies the positive or negative perceptions of individuals regarding behavior. SN refers to how social pressures can influence the performance of a certain behavior. PBC represents the person’s aspects towards difficulty level of conducting the behavior. Similar to TRA model, TPB also emphasizes that intention is the direct antecedent of behavior, and the greater intention will more likely cause behavior to be performed (Ajzen 1991). The study by Barba-Sánchez et al. (2022), considered TPB components in their study and found that PA and PBC have a direct influence on the EI of students, while SN does not influence the EI of students directly, but mediate the relationship between environmental awareness and PA; Environmental awareness and EI.

### The theory of planned behavior entrepreneurial model (TPBEM)

Based on TPB, the theory of planned behavior entrepreneurial model (TPBEM) by Krueger and Carsrud (1993) explains the three factors that impact individuals’ intentions to start a business: attitude towards venture creation, subjective norms and perceived control for entrepreneurial demeanor.

### The entrepreneurial intention model (EIM)

The entrepreneurial intention model (EIM) by Boyd and Vozikis (1994) is further developed from Bird’s (1988) original model of entrepreneurial intentionality. According to Bird’s model (1988), a person establishes intentions towards entrepreneurship based on both contextual and personal characteristics. Specifically, the contextual factors include political, social and economic elements that can shape an individual’s rational thinking. While personality, ability and background can affect one’s intuitiveness about starting a business. Based on the original model, EIM of Boyd and Vozikis (1994) added the self-efficacy factor as an application from social cognitive theory (Bandura 1977a, b, 1986) to demonstrate its importance in impacting entrepreneurial intentions and behaviors of individuals as well as being intermediary between one’s thoughts regarding entrepreneurship and intentions towards venture creation.

### The social cognitive career theory model (SCCT)

Social learning theory (SLT) was first introduced through the Bobo Doll Experiment in the 1960s by Bandura et al. (1961, 1963), indicating that learning can happen by observing, imitating, practicing behaviors and encountering consequences of behaviors in a social context (Bandura 1977a). The learning procedure is determined by the association between individuals and the degree of elevating emotional and practical expertise, defining self-perception and others’ perceptions (Bandura 1977a). Bandura (1977b) adopted the concept of self-efficacy to SLT to demonstrate the correlation between perceived self-efficacy and changes in behavior. Self-efficacy refers to a person’s belief in successfully performing a task in a certain situation. The proposed model presents four primary antecedents that develop the personal efficacy expectations, including performance achievements, vicarious experience, social persuasion, and physiological conditions, such as anxiety and arousal.

Social cognitive theory (SCT) is further developed from the SLT of Bandura, strengthening the vital role of cognitive components in the social learning process. The self-efficacy factor from his previous studies is also included in SCT as one of an individual’s behavioral determinants. Moreover, the model presents that human behavior is shaped by the reciprocal causation among environmental, behavioral, and cognitive attributes (Bandura 1986).

Lent et al. (1994) expanded the social cognitive career theory (SCCT) from the original SCT of Bandura, studying the decision-making demeanor that involves career matters. It is denoted by SCCT that career success is affected by cognitive-related elements, which consists of self-efficacy, expectancy of outcomes and intentions; and a career decision-making procedure is regulated by both personal and contextual factors.

### Lüthje and Franke model (LFM)

Lüthje and Franke Model (LFM) developed by Lüthje and Franke (2003) indicates the crucial significance of personality traits in explaining self-employment attitudes and entrepreneurial behaviors besides personal attitudes, social norms, contextual and economic determinants. Specifically, in the study concerning university's influence on the entrepreneurial intentions of students, Lüthje and Franke indicated that the components of personality traits, including risk-taking propensity and internal locus of control, have a significant influence on attitude towards entrepreneurship, hence indirectly impacting the intention to establish a new business. The model also incorporates perceived support and perceived barriers as contextual factors to determine the importance in reinforcing an individual’s intentions towards entrepreneurship (Lüthje and Franke 2003). By integrating personal and environmental factors, this model presents an extended approach to investigate the broad range of antecedents that affect entrepreneurial intentions (Nabi et al. 2010).

## Research methodology

**Fig. 1:**
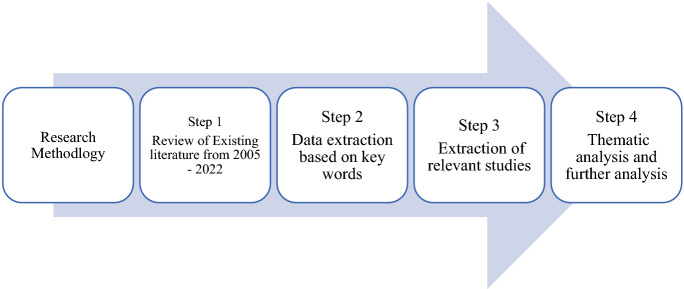
Research Methodology (adapted from Maheshwari et al. (2021))

A systematic review has been conducted for this study and the papers which measured the entrepreneurial intentions of the university students published from 2005 until June 2022 were analyzed. A four-step review was adopted for this study (Maheshwari et al. 2021). The first step focused on reviewing articles measuring the entrepreneurial intentions (EI) of students (from 2005 to June 2022). During the next step, the data extraction was conducted using various databases such as Scopus, Emerald, Springer, Taylor and Francis, ProQuest and JSTOR as many highly ranked journals of educational studies are indexed in these prominent databases and aligned with publication standards. An additional search was conducted on Google Scholar to include relevant articles. The search began using the key words such as “Entrepreneurial Intention” AND “determinants” OR “factors” AND “university students”, which resulted in 387 results (including articles, books, conference papers) in which 342 articles were in English. The third step focused on extracting the articles of our interest based on the research objective regarding factors influencing EI of students. After reading the abstract of 342 articles, 52 articles did not match our research objective and hence those were discarded leaving us with 290 final articles relevant to this study, most of which were found from the Scopus database (Table 1). During the last step, the articles were analyzed using the average citations received per year to identify the most influential papers based on the average citation per year of 10 or above. This resulted in 36 papers (Table 2) and the thematic analysis was conducted to identify the various themes from these papers regarding factors affecting EI of students. The rest of the papers (n = 254) (Table in Appendix) were analyzed further based on the identified themes. The research methodology for this paper is shown in Fig. 1.

**Table 1:** Sources of database for all 290 papers (including influential and non-influential papers)

Sources	Number of studies
Scopus	267
ProQuest	19
Google Scholar	4

**Table 2:** List of most influential papers (n = 36)

Region	Average citation per year	Name of authors	Name of journals	Database	Model used
America	80	(Zhao et al. 2005)	Journal of Applied Psychology	Scopus	SCT
Mix regions	54	(Iakovleva et al. 2011)	Education and Training	Scopus	TPB
Europe	51	(Nowiński et al. 2019)	Studies in Higher Education	Scopus	TPB; EEM; SCT
Europe	44	(Hockerts 2017)	Entrepreneurship: Theory and Practice	Scopus	TPB
Europe	38	(Maresch et al. 2016)	Technological forecasting & social change	Scopus	TPB
Mix regions	35	(Gurel et al. 2010)	Annals of Tourism Research	Scopus	Studies of Learned (1992) and TPBEM
Asia	34	(Zhang et al. 2014)	International Entrepreneurship and Management Journal	Scopus	TPB; EEM
Europe	26	(Kuckertz and Wagner 2010)	Journal of Business Venturing	Scopus	Sustainable entrepreneurship
Asia	26	(Turker and Selcuk 2009)	Journal of European Industrial Training	Scopus	Entrepreneurial support model (ESM)
Asia	26	(Al-Jubari et al. 2019)	International Entrepreneurship and Management Journal	Scopus	TPB; SDT (self-determination theory)
Europe	20	(Iglesias-Sánchez et al. 2016)	Education and Training	Scopus	TPB
Mix regions	18	(Pruett et al. 2009)	International Journal of Entrepreneurial Behaviour & Research	Scopus	None
Europe	18	(Mirjana et al. 2018)	Economic Research-Ekonomska Istrazivanja	Scopus	TPB
Mix regions	15	(Fragoso et al. 2020)	Journal of Small Business and Entrepreneurship	Scopus	TPB
Asia	15	(Wu and Wu 2008)	Journal of Small Business and Enterprise Development	Scopus	TPB
Europe	15	(Solesvik 2013)	Education and Training	Scopus	TPB
Asia	14	(Nguyen et al. 2019)	Children and youth services review	Scopus	TPB
Asia	13	(Karimi et al. 2017)	International Journal of Psychology	Scopus	TPB
Europe	12	(Maes et al. 2014)	European Management Journal	Scopus	TPB
Asia	12	(Zhang et al. 2020)	Journal of Hospitality, Leisure, Sport and Tourism Education	Scopus	Social learning theory; Positive psychology theory
Africa	12	(Hattab 2014)	Journal of Entrepreneurship	Scopus	TPB; EEM
Europe	12	(Arranz et al. 2017)	Studies in Higher Education	Scopus	TPB
Asia	11	(Mustafa et al. 2016)	Journal of Entrepreneurship in Emerging Economies	Scopus	LFM
Asia	11	(Sharahiley 2020)	Global Journal of Flexible Systems Management	Scopus	TPB; EEM
Asia	11	(Tung et al. 2020)	The international journal of management education	Scopus	Theory of reasoned action, TPB, EEM and model of entrepreneurial potential
Asia	11	(Sesen 2013)	Education and Training	Scopus	LFM
Europe	11	(Solesvik et al. 2014)	Education and Training	Scopus	Entrepreneurial event theory, cultural values theory and human capital theory
Mix regions	11	(Gieure et al. 2019)	International Journal of Entrepreneurial Behaviour and Research	Scopus	TPB
Asia	11	(Liu et al. 2019)	Frontiers in Psychology	Scopus	TPB
Asia	10	(Akhter et al. 2020)	Journal of Asian Finance, Economics and Business	Scopus	TPB
Mix regions	10	(Giacomin et al. 2011)	International Entrepreneurship and Management Journal	Scopus	None
America	10	(Martins and Perez 2020)	International Journal of Entrepreneurial Behaviour and Research	Scopus	TPB; EEM
America	10	(Zhang and Cain 2017)	International Journal of Entrepreneurial Behaviour and Research	Scopus	TPB
America	10	(Zhang et al. 2015)	Entrepreneurship Research Journal	Scopus	TPB
Asia	10	(Luc 2018)	Journal of Asian Finance, Economics and Business	Scopus	TPB
Asia	10	(Utami 2017)	European Research Studies Journal	Scopus	TPB

## Results

### Region of research

**Fig. 2:**
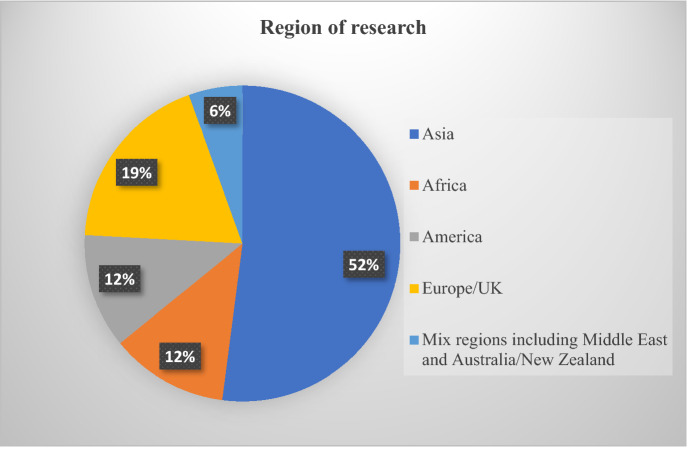
Region of research

The first criteria to analyze the paper was based on the region of research and is presented in Fig. 2. The analysis of the articles showed that most of the studies were conducted in Asia (52%), followed by 19% of studies conducted in Europe/UK region, 12% studies in both America and Africa region and the remaining 6% of the studies were multi-country studies.

### Year of research

**Fig. 3:**
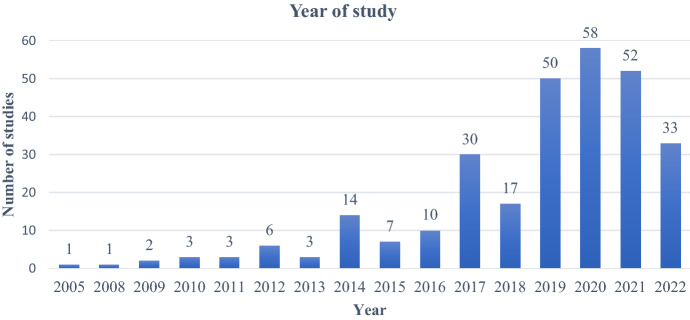
Year of study

Next, the articles were analyzed to determine the number of studies conducted in different years. It was found that very few studies had been conducted until 2016 (n = 50). Most of the studies were conducted after 2016 (n = 240). This indicates the growing interest of scholars in this area since 2017 (as in Fig. 3).

### Research methods used in the studies

Out of the 290 studies conducted between 2005 and June 2022, most of the studies (n = 285) used quantitative research methodology as seen in Table 3 and three studies used qualitative methodology. One study used mixed methodology while another study was a synthesis of literature. This clearly indicates that quantitative research methodology dominates this field of research and there is a need to use qualitative or mixed methodology approaches in this research field (Table 3).

**Table 3:** Research methods used in the studies

Research methods used	Number of studies
Quantitative	285
Qualitative	3
Mixed methodology/Literature review	2

### Citation analysis

The next stage of the analysis included the citation analysis to identify the most influential papers from the total 290 papers identified in the study. The authors analyzed all 290 final articles and calculated the average citations received per year for each paper and identified a total of 36 articles as the most influential paper based on the average citation of 10 or above received per year. These 36 most cited papers were further analyzed to identify the themes based on content similarities regarding the factors affecting the entrepreneurial intentions of university students. After reading 36 papers, the authors identified seven main themes (factors) which affect the EI of the students. The seven themes of this paper were identified based on our holistic understanding of two criteria (1) the core factors (variables) considered in all the influential papers (2) the common core factors used by these influential papers to measure the EI of students. These seven factors are cognitive factors, personality factors, environmental factors, social factors, educational factors, contextual (situational) factors and demographic factors. These factors are classified from the 36 papers identified and Table 4 shows these seven themes (factors).

**Table 4:** Themes from influential papers

Themes (in italics) and relevant factors in particular theme (non-italicized)	
*Cognitive factors*	
Cognitive styles	Self-efficacy
Personal attitude (PA)	Perceived desirability
Subjective norm (SN)	Perceived feasibility
Perceived behavioral control (PBC)	
*Personality factors*	
Risk propensity	Risk aversion
Internal locus of control	Intrinsic motivation
Creativity	Extrinsic motivation
Tolerance of ambiguity	Creativity
Leadership styles	
*Environmental factors*	
Government support	Physical infrastructure
Access to capital	Culture
Human resources	Institutional infrastructure
*Social factors*	
Prior experience	Entrepreneurial family
Role models	Culture/Country
Life experiences	
*Educational factors*	
Entrepreneurial environment of a university	Curriculum
Entrepreneurial courses	Extracurricular activities
*Contextual (situational) factors*	
Unemployment	Social status
Expected value of starting a business	Perception of support and barriers
Job dissatisfaction	Need for satisfaction
Opportunities	Desire for independence
*Demographic factors*	
Gender	Age
Nationality	Educational level
Major	

The Education and Training Journal has published the highest number of papers (five) identified in this study, followed by four published in International Journal of Entrepreneurial Behaviour and Research, three in International Entrepreneurship and Management Journal, two each respectively in Journal of Asian Finance, Economics and Business and the Studies in Higher Education. There was one article each in the rest of the journals. There are 15 studies from Asia, 10 studies conducted in Europe, six were a collaboration between multiple countries, four in America and one from Africa. 19 out of 36 studies have used the TPB model whereas seven studies have combined the TPB model with other models (as per Table 2).

### Themes (categories) identification from influential papers

The 36 most cited papers were further analyzed to identify the themes used in these papers following Fitz-Koch et al. (2018) research and it was found that most of these studies revolved around seven categories (themes). The summary of the factors under each category (theme) are summarized in Table 4. These seven identified categories (themes) used in various influential papers are discussed next in detail.

#### Cognitive factors

##### TPB factors

The effect of TPB antecedents—attitudes, social norms and perceived behavioral control—on entrepreneurial intentions is discussed in many studies, including Solesvik (2013), Zhang et al. (2015), Iglesias-Sánchez et al. (2016), Karimi et al. (2017), Mirjana et al. (2018), Al-Jubari et al. (2019). In research by Wu and Wu (2008), personal attitude is the critical influence of entrepreneurial intentions, regardless of the educational backgrounds of students. The three antecedents were proven to have a direct and significant impact on entrepreneurial intentions in the study of Solesvik (2013) and Utami (2017). Furthermore, perceived entrepreneurial motivation, which was enhanced among students who joined an enterprise education program, stimulated entrepreneurial intentions through the mediating effect of the three TPB factors (Solesvik, 2013).

Zhang et al. (2015) proved that social norms and perceived behavior control relate significantly to entrepreneurial intentions of students, and controlled behavior generates greater impact on the intentions than social norms do. However, no relationship is found between attitudes and the desire to start a business, considering the lack of experience towards entrepreneurship among undergraduate students. In the research of Iglesias-Sánchez et al. (2016), PA and PBC have a significant impact on students’ intentions to start ventures, while SN is not a determining component but only demonstrates a decision-making role. In line with other studies, Karimi et al. (2017) confirmed the significant impact of TPB antecedents on Iranian students’ EI. Among the three predictors, PBC shows the strongest relationship with intentions to start a business while SN demonstrates the weakest. Maresch et al. (2016) highlighted a negative relationship between subjective norms and entrepreneurial intentions for science and engineering students. By applying TPB to investigate entrepreneurial intentions of students between developing and developed countries, Iakovleva et al. (2011) showed stronger entrepreneurial intentions with higher attitudes, SN and PBC in developing countries. The result of the study indicated that SN significantly impacted entrepreneurial intentions of students, which is different from some past papers that found no significance in the relationship between SN and intentions towards entrepreneurship (Chen et al. 1998; Wu and Wu 2008).

##### Other factors

The study of Zhang et al. (2014) identified that entrepreneurial intentions are significantly influenced by perceived desirability, but not by perceived feasibility. This is explainable by negative environmental elements of perceived behavioral control, uncertain locus of control and environmental controls due to lack of prior experiences. Solesvik et al. (2014) stated that in a transitional economy context, students that have perceived desirability and perceived feasibility have greater entrepreneurial intentions. Moreover, the study found that students who take initiative have greater intentions to start a business, while students with low capability beliefs yield lower entrepreneurial intentions. The research of Mirjana et al. (2018) also highlighted the crucial relationship between innovative cognitive style and students’ intentions to become entrepreneurs. However, innovative cognitive style inconsiderably influences entrepreneurial intentions when the factor is considered as a solely explanatory component.

#### Personality factors

According to Zhao et al. (2005), people having higher risk propensity will be more confident confronting risky situations and viewing uncertain circumstances as less risky than other individuals. Considering that, they might feel less anxious to take on entrepreneurial occupation, fulfill the position and complete the tasks more comfortably, thus having higher entrepreneurial self-efficacy. This impact is also justified by the findings of the study, stating that self-efficacy plays a mediating role in the relationship between risk propensity and intentions towards entrepreneurship of students.

Many studies found that self-efficacy and intentions towards entrepreneurship of students have a positively significant relationship, such as Guzmán-Alfonso and Guzmán-Cuevas (2012), Sesen (2013), Utami (2017), Zhang and Cain (2017). The research of Gurel et al. (2010) indicates that innovativeness and risk-taking propensity play a significant role as a predictor for entrepreneurial intentions of tourism students in the UK and Turkey, while tolerance of ambiguity and locus of control do not relate to the intentions towards entrepreneurship. Additionally, education does not demonstrate the moderating impact in the relationship between those entrepreneurial traits and intentions of tourism students. On the other hand, locus of control shows significant influence on entrepreneurial intentions in the study of Sesen (2013). The study also indicates that personality factors are more significant towards the entrepreneurial intentions of students than environmental predictors such as access to capital and social networks.

Zhang et al. (2015) also stated that short-term risk-taking preference and factors of psychological well-being positively influence the intentions towards entrepreneurship of an individual with the existence of TPB antecedents, namely personal attitudes, social norms and perceived behavioral control. The study of Zhang and Cain (2017) pointed out that there is an absence of a direct effect of risk aversion on intentions to start a business in dental school students. Risk aversion only diminishes entrepreneurial intentions indirectly via antecedents of TPB. The impact stated that risk aversion might not be a fixed dispositional factor, but an adjustable trait that can be altered over time. The study of Mustafa et al. (2016) found that entrepreneurial intentions are positively impacted by a proactive personality and the perceived university support of Malaysian students. Among the two drivers, proactive personalities have a stronger influence on intentions towards self-employment than from the perceived concept of student development support from students. Karimi et al. (2017) stated that personality factors, including the need for achievement, risk taking and locus of control, indirectly affect the intentions towards entrepreneurship of Iranian students through the entrepreneurial attitudes and PBC.

In the paper of Al-Jubari et al. (2019), intrinsic and extrinsic motivations, which are generated from psychological needs of autonomy, relatedness and competence, are indicated to have a positive indirect effect on entrepreneurial intentions in Malaysian students via the three components of TPB. More importantly, the motivation types play important roles in the entrepreneurial process, in which each kind yields different impacts. Entrepreneurs with intrinsic motivations will demonstrate more effective performances, more persistence and greater autonomy, leading to dynamic entrepreneurs. Meanwhile, individuals with extrinsic motivations will be less persistent when confronting challenges, more likely to discontinue nascent behaviors, and concentrate on external achievements. In addition, the need for satisfaction and the need for frustration are also proven to be negatively correlated.

#### Environmental factors

In the study, Pruett et al. (2009) indicated that the participants’ country and cultures, exposure to acquaintance entrepreneurs in family, family support, and the strength of beliefs towards motives for entrepreneurship positively impact entrepreneurial intentions. However, the significance of those variables is smaller than the effect of entrepreneurial disposition, implying that factors related to personal characteristics are more influential on the intentions towards self-employment. In addition, entrepreneurial intentions are negatively affected by social barriers, which involve operating risks, or lack of knowledge and capital. But the impact is also relatively small as compared to the disposition factor.

According to Turker and Selcuk (2009), structural support shows significant impact on entrepreneurial intentions, stating that a greater comprehensive support from all social sectors is required to stimulate entrepreneurship in young people. In addition, the effect of self-confidence as a moderating component is more considerable in the connection between structural support and entrepreneurial intentions. However, the study demonstrates that perceived relational support, such as monetary and sentimental assistance from family and friends, does not influence the intention to establish a business for students. Access capital is proven to have a negative and significant correlation to entrepreneurial intentions of students in the study of Sesen (2013).

#### Social factors

Prior entrepreneurial experience, which provides students a role model and enactive proficiency from empirical exposure, is proven to be strongly mediated by self-efficacy, thus further affecting entrepreneurial intentions (Zhao et al. 2005). However, prior entrepreneurial exposure shows a notable negative impact on intentions towards entrepreneurship in the research of Zhang et al. (2014). The reason for this unexpected result is that the participants in the study mostly underwent negative entrepreneurial experiences, thus raising the fears and insecurities towards self-employment. The proposed model of Kuckertz and Wagner (2010) indicated the orientation of an individuals' sustainability is meaningful in the relationship with their entrepreneurial intentions. Nevertheless, while significant potential is found among students for entrepreneurship opportunities with sustainable orientation, it declines as business experience is achieved.

Gurel et al. (2010) included social factors into the research model to explain the effect on entrepreneurial intentions of tourism students. Among those elements, students with entrepreneurial families tend to have higher intentions towards entrepreneurship. Conversely, cultural factors are considered when there is concern in the probability of self-employment, instead of entrepreneurial intention. The findings of Hockerts (2017) showed that prior experience with social organizations can increase social entrepreneurial intentions of an individual. The relationship is mediated by factors including empathy, self-efficacy, moral obligation, and perceived social support. The self-efficacy variable holds the greatest effect. Furthermore, the study provided compelling evidence that the number of social entrepreneurship electives by students is predicted by entrepreneurial intentions.

#### Educational factors

The perceptions of formal learning from students does not directly influence entrepreneurial intentions but demonstrates a strong indirect impact on the decision of an individual to start a business via a mediating factor—self-efficacy. Supporting the idea that students’ intentions towards entrepreneurial venture creation can be shaped by formal academic courses. The study of Zhao et al. (2005) suggests educational institutions integrate distinct types of learning ways to improve entrepreneurial self-efficacy in students. Entrepreneurial intentions of students are proven to be impacted at educational level through personal attitude effects and academic majors through both attitudes and the PBC of individuals. However, no relationship is found between academic accomplishment and PBC; and entrepreneurship education curriculums have little to no impact on the entrepreneurial desires of students (Wu and Wu 2008). One highly influential paper by Walter and Block (2016) identified the effects of entrepreneurship education is higher on the EI of students in entrepreneurship-hostile institutional environments than in entrepreneurship-friendly institutional environments. Although the paper was highly cited, we could not include it in this list because it was based on macrolevel educational factors, whereas this study is based on microlevel educational factors.

The result from the study of Turker and Selcuk (2009) indicates that educational support significantly impacts the entrepreneurial intentions of students in Turkey. As specified by the paper, an educational institution delivering sufficient knowledge and motivation towards entrepreneurship to students will enhance the likelihood of young people being involved in venture creation, thus suggesting universities to develop educational policies and structures to effectively inspire entrepreneurs. Nevertheless, self-confidence, which is a moderator in the model, was proven to not strengthen the relationship between educational support and intentions towards entrepreneurship. Moreover, according to the article, the educational factor has greater beta co-efficiency than structural support factor, the former variable is a more influential predictor to entrepreneurial intentions than the latter. This can be explained that students might perceive educational support as an immediate factor, thus having more awareness towards this support (Turker and Selcuk 2009).

According to the study of Arranz et al. (2017), although curricular and extracurricular activities have a positive impact on attitudes towards entrepreneurship, they might reduce capacity and intention to engage in the start-up activities of students. Moreover, the research demonstrates different influences of curricular activities on the entrepreneurial competences of students in two institutions, suggesting the development in educational methodology and strategies to enhance their competences. There are a substantial number of studies indicating the positive connection between entrepreneurship education and entrepreneurial intention of students (Hattab 2014; Zhang et al. 2014; Maresch et al. 2016; Nowiński et al. 2019). Research from Zhang et al. (2014) shows a direct relationship between entrepreneurship education and entrepreneurial intentions. Also, gender, study majors and university types have considerable positive interactive impacts on the correlation between entrepreneurship education and intentions to run a business in the context of Chinese university students. The findings of Hattab (2014) and Maresch et al. (2016) are also in line regarding the positive impact of entrepreneurship education towards entrepreneurial intentions of students. It is noteworthy that students in business majors may benefit more from entrepreneurship education than those in science and engineering programs. In the study of Hattab (2014), education is indicated to positively influence perceived desirability towards entrepreneurship of students, while the effect is less significant on perceived feasibility and inconsiderable on students’ self-efficacy. The positive significant relationship between entrepreneurship education and entrepreneurial intentions, mediated by self-efficacy, is also confirmed in the study of Nowiński et al. (2019). Among Visegrad countries, the correlation is considerable only in Poland where entrepreneurship education is introduced in high school. Another important finding is that despite lower levels of self-efficacy and intentions towards entrepreneurship in female students, they may benefit more from entrepreneurship education than males do. However, some studies found no relationship between university environment and entrepreneurial intentions of students, including the work of Sesen (2013), Chen et al. (2015).

According to Chen et al. (2015), entrepreneurship courses increase learning efficacy and satisfaction of technical undergraduate students, but do not enhance the intentions of students to start a business. The study implies that entrepreneurship courses provided by universities would help students to recognize that entrepreneurial occupations might not be suitable for them and rather to implement what they learned to future jobs rather than pursue entrepreneurship. Solesvik et al. (2014) proved that although the relationship between students joining in entrepreneurship-specific education and high intensity of entrepreneurial intentions is positive, the interactions of entrepreneurship-specific education with perceived desirability, perceived feasibility, and perceived cultural elements are not related to greater entrepreneurial intentions.

#### Contextual (situational) factors

According to Guzmán-Alfonso and Guzmán-Cuevas (2012) perceived social value and entrepreneurial intentions have a significantly negative relationship for people aged 18–64 in Latin America, which is not in line with Ajzen's model. The study of Karimi et al. (2017) indicated that perceived contextual support and barriers have an indirect impact on entrepreneurial intentions of students via PBC; and perceived barriers are also found to have a direct, negative association with the intentions towards start-up. The entrepreneurial intentions were found to be positively related to perception of motives such as creativity and desire for independence while perceptions of barriers were negatively impacting the entrepreneurial intentions of the students as mentioned by Pruett et al. (2009) in their study.

#### Demographic factors

##### Gender differences

Gender was proven to have a direct relationship with entrepreneurial intentions in the study of Zhao et al. (2005), in which females show lower intentions to start a business than males. However, the research indicates that there is no difference between the two genders regarding entrepreneurial self-efficacy, suggesting that the connection between sexuality and entrepreneurial intentions is shaped by theoretical mechanisms such as perceived social supports and barriers, and outcome expectations, rather than self-efficacy. Empirical results from Zhang et al. (2014) state that women and people from universities and backgrounds, other than technology, have lower entrepreneurial intentions than men and people from technological ones, suggesting eliminating the traditional entrepreneurial stereotypes among females and increase the need for additional women role models. Furthermore, the research of Maes et al. (2014) investigated the differences between females and males in the elements that predict entrepreneurial intentions of graduate students in Belgium. Females tend to be driven to self-employment by the motivation of getting organized and in consideration of their own personal abilities, while males will base their entrepreneurial intentions on financial restraints and creativity while perceiving entrepreneurship as a means of getting ahead. In addition, females prefer to comply with normative role models than males; however, the gender impact on entrepreneurial intentions is not mediated by social norms.

##### Nationality differences

The study of Giacomin et al. (2011) determined the national differences in entrepreneurial intentions, motivations, and perceived barriers towards venture creation dispositions of students in American, Asian, and European countries. Specifically, the strongest entrepreneurial intentions are found in Spanish students, while interests towards public administration occupations are identified in Chinese students. Furthermore, despite similarly perceived motivations and barriers to self-employment, students in those countries show different sensitivity levels and significant extents to each motivator and barrier, which can be explained by the socio-economic factors of each nation.

Regarding students in Beira Interior region (Portugal) and Extremadura region (Spain), there is evidence that some differences exist in perceived desirability, perceived feasibility, and entrepreneurial intentions among the countries according to the study of Díaz-Casero et al. (2012). University students in the two nations have positive perceptions towards the desirability of entrepreneurship. Concerning perceived feasibility, Spanish students found it easier to start a business in the present than in the past, while this was not true for Portuguese students. Moreover, students in the Extremadura region have higher intentions to start a business than those in the Beira Interior region; nevertheless, the seriousness of entrepreneurial intentions is higher in Portuguese students compared to individuals in Spain. Additionally, the influences of gender and entrepreneurial family members on the perceptions of students in the two countries are also investigated, in which sexuality impacts the perceived intentions of students. Yet no influence is found between gender, family backgrounds and perceived feasibility in both groups.

### Further analysis of themes from influential papers

**Fig. 4:**
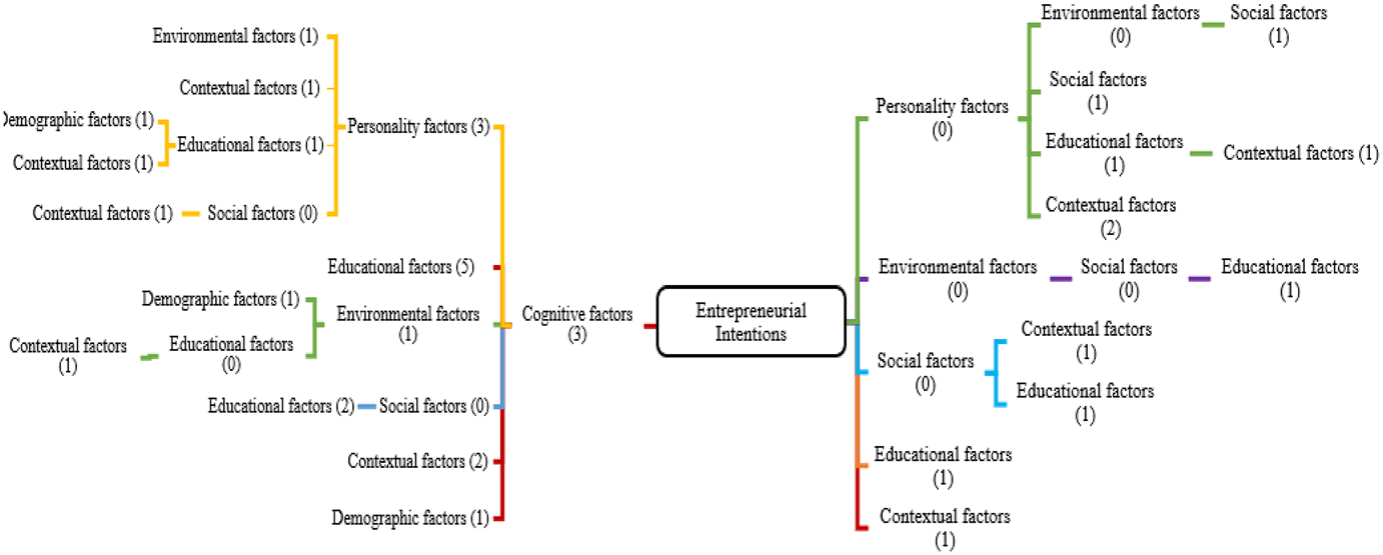
Mind map showing most common factors in (n = 36) most influential papers. *Note*: Mind map reading; for example: EI affected by cognitive factors (3 studies) (on left of EI box); EI affected by cognitive factors and personality factors (3 studies) (on left of EI box); EI affected by cognitive, personality and environmental factors (1 study) (on left of EI box); EI affected by cognitive factors, environmental factors, demographic factors (1 study) (on left of EI box); EI affected by only education factors and contextual factors (1 study) (on right of EI box); EI affected by personality factors and educational factor (1 study) (on right of EI box)

To visually present the themes clearly, the mind map has been used (as in Fig. 4) to summarize as what factors are most commonly considered in these 36 most influential papers and the analysis suggests that cognitive factors are most commonly used (three studies used them as only factors affecting entrepreneurial intentions) or cognitive factors used with personality factors (in three studies) or cognitive factors with educational factors (used in five studies) or cognitive factors used with environmental factors, demographic factors and contextual factors respectively in one, one and two studies. This shows that most of the papers (15 out of 36) have used cognitive factors as only factors or combined with other factors affecting EI of students. Personality factors, environmental factors or social factors are never considered alone in these most cited papers but used in combination with other factors and that too are not very widely used. Other factors, such as social factors, educational factors, contextual factors, and environmental factors, are rarely used as single factors to measure the EI of students.

**Fig. 5:**
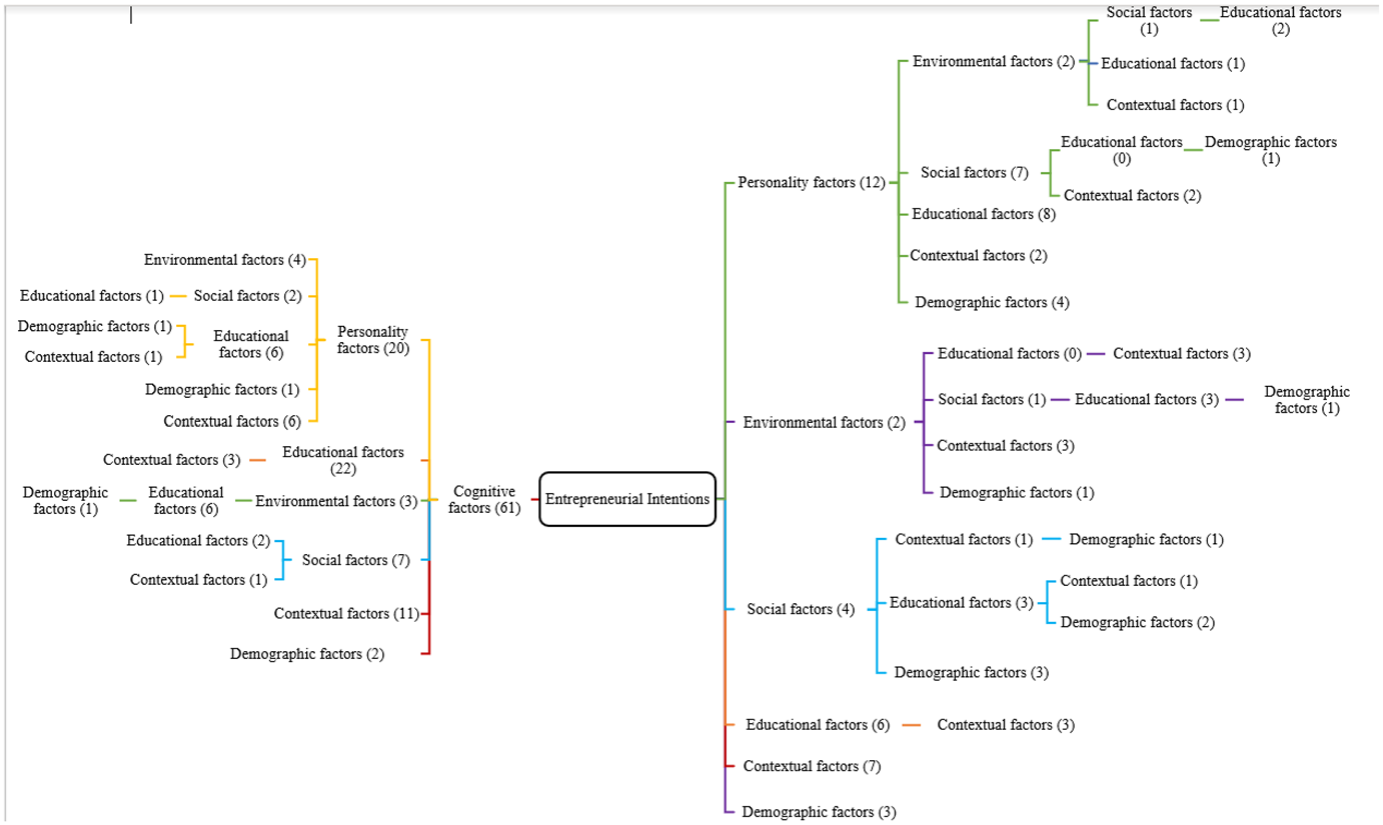
Mind map showing most common factors in n = 254 papers (non-influential papers)

Based on these identified seven-factors themes, the rest of the 254 papers were also grouped in similar way as seen in Fig. 5. This analysis also indicates that the cognitive factors are used as a single factor in 61 studies, followed by a combination of cognitive and personality factors by 20 studies, cognitive and educational factors by 22 studies, cognitive and contextual factors by 11 studies and cognitive factors with social, environmental, demographic factors considered by respectively seven, three and two studies. Overall, 161 studies used cognitive factors (single or in combination with other six factors), followed by 43 studies which used personality factors alone or in combination with the rest of the five factors. The pattern found in the rest of the papers are like the ones used by most cited papers except that in these papers, the personality factors are explored in more detail.

### Conceptual model

Based on the synthesis of literature, we propose the integrated conceptual model of the variables that affects the entrepreneurial intentions of the students according to the extensive coding of 290 papers. The first important finding is that the independent variables used to measure the entrepreneurial intentions of university students are captured in the literature at seven levels: cognitive factors, personality factors, environmental factors, social factors, educational factors, contextual factors and demographic factors. Most of the papers have used cognitive factors (TPB model, EEM, EIM) as an independent variable and TPB components are used as mediators/moderators to measure the EI of students.

The second important finding that the model depicts is that there are a wide range of mediators and moderators used to measure the EI of students. The mediators and moderators are also measured at the same seven levels as mentioned for independent variables. In total, 130 studies used mediators and 61 scholars used moderators in their study. Most of the studies have used TPB components as mediator, while moderators used are mostly related to demographic factors, contextual factors, and personality factors.

**Fig. 6:**
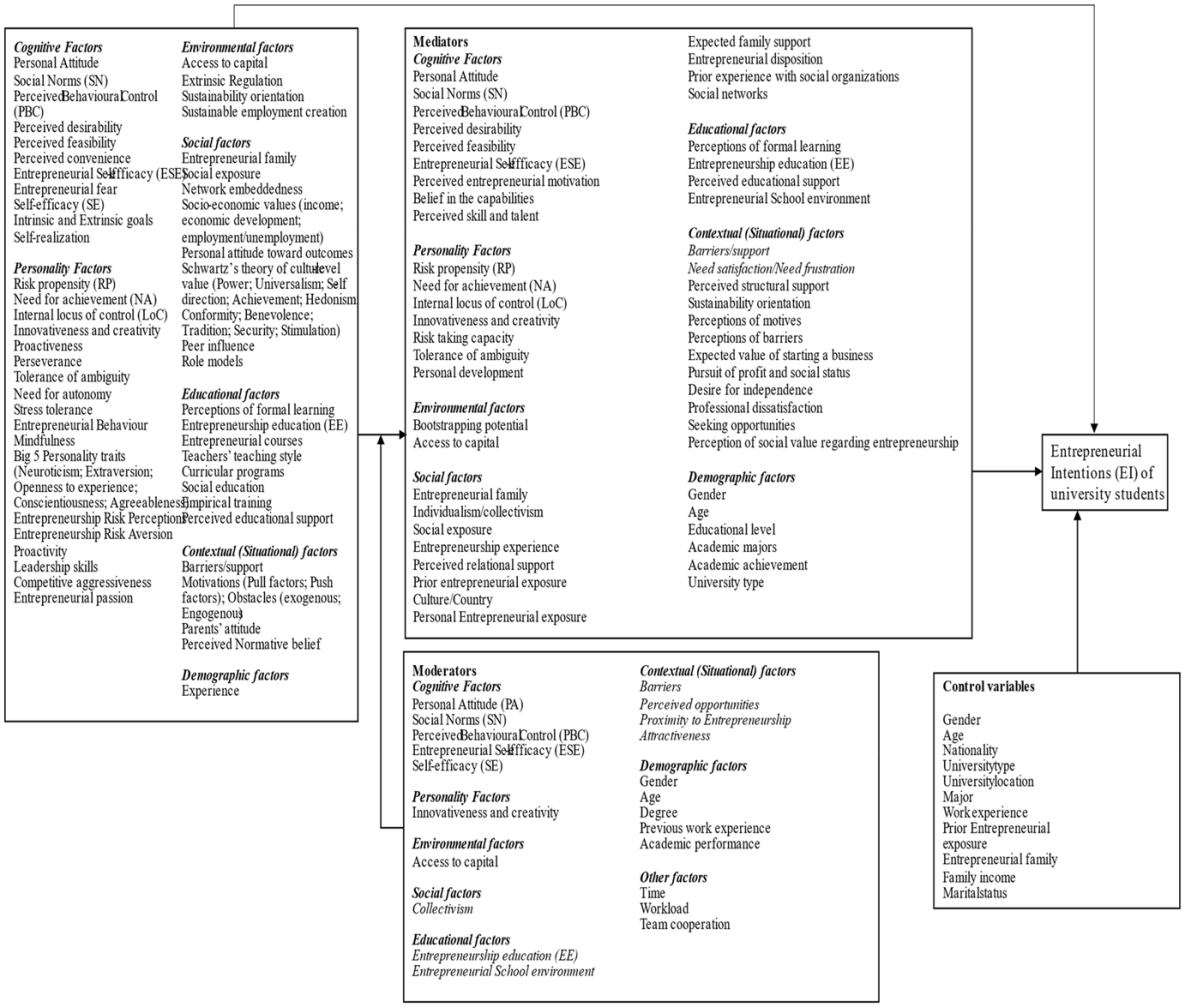
Conceptual model (Factors influencing EI)

Other than TPB factors, examples of mediators used as cognitive factors from entrepreneurial event model are perceived desirability, perceived feasibility, and entrepreneurial self-efficacy. Other mediators used are risk propensity, need for achievement, and locus of control which are derived from Bandura’s social cognitive theory and are related to personality factors. The next level of mediators used are at a social level such as the family influence, social exposure, country culture, and prior experience. The next majorly used mediators are related to situational factors as shown in conceptual model, followed by educational factors, and demographic factors. Only 21% of scholars in their studies have used moderators. The moderators also consist of seven identified factors (see Fig. 6). Some of the scholars have also used control variables in their study which are mostly demographic variables such as gender, university type, university locality, nationality, major, age, work experience, and exposure to entrepreneurial activities, family income, marital status, and entrepreneurial family background.

## Discussion

Entrepreneurship studies have arisen rapidly since the published works of Shapero 40 years ago (Shapero and Sokol 1982; Shapero 1984), many research papers have focused on EI, which is considered to have the greatest influence on entrepreneurship activities. This paper systematically analyzed various publications regarding the EI of students during the period 2005 till June 2022 and discusses the most and common studied factors in past research and how future studies can further explore this research area.

The paper adopted citation analysis, which is a prominent method, that recognizes the 36 most impactful studies in this research area over the period considered in this study. From the analysis, seven main themes (categories) of EI determinants were identified and implemented further in the paper’s analysis framework to analyze the rest of the articles (n = 254) used in this study. The analysis on several factors from 254 studies is briefly included in this review paper and indicated similar results as found in most influential papers, which suggests that the cognitive factor is most used factor in many papers which influences the EI of students.

### Theoretical implications and suggestions on future research

From a theoretical perspective, the literature review aims to identify the factors most studied by past scholars that have an impact on the EI of university students. The study contributes to the educational entrepreneurship research literature, which will help higher educational institutions to understand which significant factors stimulate students’ intentions to start a business. The analysis of the paper clearly demonstrated that TPB model and cognitive factors dominate this area of research, and most studies are found to be conducted in Asian countries. Hence, based on the analysis of papers, this study discusses further steps for entrepreneurship research to better understand EI of university students and offers the following suggestions as below and as summarized in the conceptual model for future research (Fig. 6).

It is clear that to improve the understanding of factors affecting entrepreneurial intentions, future research should consider moving from cognitive factors using the TPB model. It is important to access the role of social factors including the family background and culture, contextual (situational) factors as to what forces an individual to become an entrepreneur, and this may shed further light on the EI of students. These combined factors will help in bringing out a holistic overview of factors affecting students EI. Cognitive factors have been extensively used in literature and there is a need to identify other important factors which affect the EI of students. Next, the culture of the country can have a great influence on the intentions of the students which has been less explored in the studies and hence conducting research considering the socio-cultural factors in different countries might help in providing multi-faceted views in terms of entrepreneurial intentions. It can also be of interest for the scholars to further explore the demographic factors as very few studies have considered them to determine the EI of students. The outbreak of COVID-19 has affected entrepreneurship (Yu et al. 2021), and hence future research may consider the impact of the pandemic, such as online education, macroeconomic factors, etc., on EI of university students.

In the digital transformation economy, technological entrepreneurship started to grab the attention of many countries, especially developing countries (Nathani and Dwivedi 2019). The perception towards technology growth can influence students’ intentions to start new ventures. Future research can study the technological entrepreneurship by investigating how combined factors such as environmental factors (e.g., access to technology), contextual factors (e.g., perception of recent economy/market, perception of government support, opportunities), and social factors (e.g., prior experiences, role models) affect the EI of university students, from which many implications can be undertaken to benefit the young people’s entrepreneurial activities. Most of the studies provided comparisons on EI considering different nationalities or genders. Furthermore, to enhance understanding in the EI research area, comparison can be provided considering other demographic factors, for instance, online versus physical educational environment, individuals with disabilities versus the ones without disabilities. Most of the studies have focused on theory of the planned behaviour model and hence it might be important in the future to combine different EI models to explore and broaden the scope of literature which will further add value and contribute to the already existing literature.

More than half of the studies are conducted in Asia and hence it is important to explore other less explored regions such as Africa, America, and Europe to determine if there are any differences in factors affecting the EI of students in these developed and developing regions. As identified by the review, the entire literature in this field is dominated by quantitative studies and hence to provide more robust results, mixed studies using quantitative and qualitative should be conducted in the future. Future research may benefit from EI determinants in the context of higher education to conduct studies on different developmental phases, such as primary and secondary school. In the study of Brüne and Lutz (2020), entrepreneurship education had greater positive influence on the self-efficacy and entrepreneurial desirability of younger students than older ones. Scholars can examine the effect of early exposure to entrepreneurship on the development of factors that will further impact EI of students. The research of Barth and Muehlfeld (2021) found that the interventions in early entrepreneurship enhanced the entrepreneurial self-efficacy of university students.

### Practical implications

There are some critical implications based on the analysis from this paper for various stakeholders. The systematic review can be useful for the scholars who aim to conduct research in EI of students in the future and this review will inspire and motivate the scholars to determine the novel research framework based on the insights provided from this systematic review which can help the community in general. Policy practitioners can implement relevant policies and provide appropriate support to enhance the EI of individuals and to provide an environment to build an entrepreneurship culture in their country. Educational institutions and teachers can find ways to inspire the entrepreneurial intentions in the students by enhancing course curriculums, developing applicable skills and knowledge, encouraging ideas, and boosting self-confidence in students and helping them to develop overall. Entrepreneurial intentions are often used as a proxy for behavior, but entrepreneurial intentions rarely convert into entrepreneurial action, particularly among students who have limited experience of entrepreneurship and lack experience of work altogether. Therefore, focusing on entrepreneurial intentions only, or using intentions as a proxy for action, represents a severe limitation to entrepreneurial action and future studies should focus on measuring the entrepreneurial actions with entrepreneurial intentions.

## Conclusion

This paper has conducted the systematic review on more than 15 years of EI research studied in worldwide university students to provide an understanding of the most common factors that affect students’ intentions to become entrepreneurs used in the literature and suggest novel lines of research in the future. Various papers published between 2005 and June 2022 measuring the EI of the students were analyzed and the findings clearly suggest that there is an increasing trend of these studies from 2017 onwards. Most of the research focused on Asia with more than half of the studies conducted in this region. The results highlighted that the most scholars used TPB model to measure EI of students, and the factors used by most papers are related to cognitive factors. It is believed that using a range of factors can provide a better understanding to measure the EI of students. Hence, future research can be extended in various areas as identified by the gaps provided in the discussion section above. Based on the above findings, it is clear that cognitive factors should not only be accounted for to understand the intentions of students and more studies should focus on other factors which are equally important in their influence on the EI of the students.

Like every other study, this review also has some limitations that can be addressed in future research. First, although the authors tried their best to include the studies between 2005 and June 2022, some studies might have been overlooked due to the variety of databases available. Next, the comparative review between different regions might have helped to understand the region-specific factors affecting the EI of students which was not explored in this study. Next, specific review can be conducted in Africa to better understand the EI of students as there are fewer studies currently in this region and such review can motivate the scholars to explore this region further. Finally, the comparative review between Asia and Africa might provide valuable insight on the factors affecting the EI of students, as both the regions mostly consist of developing countries.

## Data Availability

Data sharing is not applicable as no datasets are generated in this current study. All the articles used for the analysis are already provided in the article (in Table 2 and Appendix table).

## Appendix

**Table:** List of n = 254 papers (non-influential papers)

Title of the paper	Name of authors	Name of Journals	Database
Agro-Entrepreneurial Intention among University Students: a study under the premises of Theory of Planned Behavior	(Che Nawi et al. 2022)	SAGE Open	Scopus
Attitudes, subjective norms, and perceived control versus contextual factors influencing the entrepreneurial intentions of students from Poland	(Kobylińska 2022)	WSEAS Transactions on Business and Economics	Scopus
COVID-19 pandemic and entrepreneurial intention among university students: a contextualisation of the Igbo Traditional Business School	(Godswill Agu et al. 2022)	African Journal of Economic and Management Studies	Scopus
Determinant factors in entrepreneurial intention among Social Work degree students: the moderating effect of entrepreneurship education	(García-Uceda et al. 2022)	Social Enterprise Journal	Scopus
Determinants of Entrepreneurial Intentions of Youth: the Role of Access to Finance	(Rusu et al. 2022)	Engineering Economics	Scopus
Drivers of Green Entrepreneurial Intention: Why Does Sustainability Awareness Matter Among University Students?	(Prabowo et al. 2022)	Frontiers in Psychology	Scopus
Effects of Entrepreneurial Competence and Planning Guidance on the Relation Between University Students’ Attitude and Entrepreneurial Intention	(Ferreira et al. 2022)	Journal of Entrepreneurship	Scopus
Factors That Can Promote the Green Entrepreneurial Intention of College Students: A Fuzzy Set Qualitative Comparative Analysis	(Cai et al. 2022)	Frontiers in Psychology	Scopus
Factors that influence the entrepreneurial intention of psychology students of the virtual modality [Factores que influyen en la intención emprendedora de estudiantes de psicología de la modalidad virtual]	(Valencia-Arias et al. 2022)	Retos(Ecuador)	Scopus
Positive psychological capital and university students’ entrepreneurial intentions: does gender make a difference?	(Maslakçı et al. 2022b)	International Journal for Educational and Vocational Guidance	Scopus
Relationship between Positive Psychological Capital and Entrepreneurial Intentions of University Students: The Mediating Role of Entrepreneurial Self-efficacy	(Maslakçı et al. 2022a)	World Journal of Entrepreneurship, Management and Sustainable Development	Scopus
Sustainable Economic Development Through Entrepreneurship: A Study on Attitude, Opportunity Recognition, and Entrepreneurial Intention Among University Students in Malaysia	(Wiramihardja et al. 2022)	Frontiers in Psychology	Scopus
Sustainable entrepreneurial intentions: Exploration of a model based on the theory of planned behaviour among university students in north-east Colombia	(Romero-Colmenares and Reyes-Rodríguez 2022)	International Journal of Management Education	Scopus
The entrepreneurial intention of university students: An environmental perspective	(Barba-Sánchez et al. 2022)	European Research on Management and Business Economics	Scopus
The impact of entrepreneurship education and cultural context on entrepreneurial intentions of Ukrainian students: the mediating role of attitudes and perceived control	(Mykolenko et al. 2022)	Higher Education, Skills and Work-based Learning	Scopus
UPPS impulsivity, entrepreneurial self-efficacy and entrepreneurial intentions among university students: ADHD symptoms as a moderator	(Tran et al. 2022)	Journal of Applied Research in Higher Education	Scopus
The Effect of Entrepreneurial Education and Culture on Entrepreneurial Intention	(Kayed et al. 2022)	Organizacija	ProQuest
Assessing the effectiveness of entrepreneurship education in the universities of Tehran province based on an entrepreneurial intention model	(Sherkat and Chenari 2022)	STUDIES IN HIGHER EDUCATION	Taylor and Francis
The influence of entrepreneurship education on entrepreneurial intentions: Perception of higher business education graduates	(Magasi 2022)	International Journal of Research in Business and Social Science	ProQuest
Determinants of Somali Student's Entrepreneurial Intentions: The Case Study of University Students in Mogadishu 1	(Alin and Dil 2022)	Eskisehir Osmangazi Universitesi Sosyal Bilimler Dergisi	ProQuest
Testing the Influence of Self-Efficacy and Demographic Characteristics among International Students on Entrepreneurial Intention in the Context of Hungary	(Wu et al. 2022)	Sustainability; Basel	ProQuest
Entrepreneurial Intention of Chinese Students Studying at Universities in the Community of Madrid	(Lin et al. 2022)	Sustainability; Basel	ProQuest
Entrepreneurship or Employment? A Survey of College Students’ Sustainable Entrepreneurial Intentions	(Zhu et al. 2022)	Sustainability; Basel	ProQuest
Drivers of Student Entrepreneurial Intention and the Moderating Role of Entrepreneurship Education: Evidence from an Indian University	(Akhtar et al. 2022)	Discrete Dynamics in Nature and Society	ProQuest
Sustainability at Universities as a Determinant of Entrepreneurship for Sustainability	(Fanea-Ivanovici and Baber 2022)	Sustainability; Basel	ProQuest
Impact of attitude towards entrepreneurship education and role models on entrepreneurial intention	(Amofah and Saladrigues 2022)	Journal of Innovation and Entrepreneurship	ProQuest
People-Centered Entrepreneurship: The Impact of Empathy and Social Entrepreneurial Self-efficacy for Social Entrepreneurial Intention	(Kim 2022)	Global Business & Finance Review	ProQuest
The Impact of Personal Values and Attitude toward Sustainable Entrepreneurship on Entrepreneurial Intention to Enhance Sustainable Development: Empirical Evidence from Pakistan	(Yasir et al. 2022)	Sustainability; Basel	ProQuest
Entrepreneurship and Sustainable Development Goals: A Multigroup Analysis of the Moderating Effects of Entrepreneurship Education on Entrepreneurial Intention	(Ashari et al. 2022)	Sustainability; Basel	ProQuest
Entrepreneurial Intention of Students (Managers in Training): Personal and Family Characteristics	(Dragin et al. 2022)	Sustainability; Basel	ProQuest
The Role of Psychological Factors on Entrepreneurial Intentions among Business Students	(Qudus et al. 2022)	Journal of Behavioural Sciences	ProQuest
Sustainability orientation and sustainable entrepreneurship intention: the mediating role of entrepreneurial opportunity recognition	(Bapoo et al. 2022)	Academy of Entrepreneurship Journal	ProQuest
Technopreneurial Intentions: The Effect of Innate Innovativeness and Academic Self-Efficacy	(Salhieh and Al-Abdallat 2022)	Sustainability; Basel	ProQuest
“Is Old Gold?” the Role of Prior Experience in Exploring the Determinants of Islamic Social Entrepreneurial Intentions: Evidence from Bangladesh	(Ashraf 2021)	Journal of Social Entrepreneurship	Scopus
Bridging the gap in real estate enterprise: the impact of mentoring on entrepreneurial intentions of real estate students in Nigeria	(Ayodele et al. 2021)	Property Management	Scopus
Determinants of entrepreneurial intention in Mexican university students [Determinantes de la intención emprendedora en estudiantes universitarios mexicanos]	(Virginia Guadalupe et al. 2021)	Revista de Ciencias Sociales	Scopus
Does the Support System Mediate the Relationship between University Roles and Entrepreneurial Intentions among University Students?	(Shamsudin et al. 2021)	Journal of Information Technology Management	Scopus
Effect of entrepreneurship education on entrepreneurial intention among university students	(Abdullahi et al. 2021)	Journal of Technical Education and Training	Scopus
Effect of gender role identity on the entrepreneurial intention of university students	(Datta et al. 2021)	Journal of Small Business and Entrepreneurship	Scopus
Entrepreneurial ecosystem, entrepreneurial self-efficacy, and entrepreneurial intention in higher education: Evidence from Saudi Arabia	(Elnadi and Gheith 2021)	International Journal of Management Education	Scopus
Entrepreneurial Self-Efficacy Mediates the Impact of the Post-pandemic Entrepreneurship Environment on College Students’ Entrepreneurial Intention	(Zhang and Huang 2021)	Frontiers in Psychology	Scopus
Entrepreneurship education and its influence on higher education students’ entrepreneurial intentions and motivation in Portugal	(Mónico et al. 2021)	BAR—Brazilian Administration Review	Scopus
Entrepreneurship education, academic major, and university students’ social entrepreneurial intention: the perspective of Planned Behavior Theory	(Chang et al. 2021)	Studies in Higher Education	Scopus
Exploring the impact of studying abroad in hungary on entrepreneurial intention among international students	(Wu and Rudnák 2021)	Sustainability (Switzerland)	Scopus
Gender, risk-taking and entrepreneurial intentions: assessing the impact of higher education longitudinally	(Gurel et al. 2021)	Education and Training	Scopus
Impacts of architectural education on entrepreneurial intention: a case study of senior architects from six universities in Turkey	(İlerisoy et al. 2021)	Archnet-IJAR	Scopus
The Antecedents of Saving Behavior and Entrepreneurial Intention of Saudi Arabia University Students	(Alshebami and Seraj 2021)	Educational Sciences: Theory and Practice	Scopus
The differential impact of the experiential-entrepreneurial learning method on the entrepreneurial intentions of higher education students	(Ali and Negasi 2021)	International Journal of Learning, Teaching and Educational Research	Scopus
The impact of entrepreneurial passion on the entrepreneurial intention; moderating impact of perception of university support	(Anjum et al. 2021b)	Administrative Sciences	Scopus
The impact of narrow personality traits on entrepreneurial intention in developing countries: A comparison of Turkish and Iranian undergraduate students using ordered discrete choice models	(Çelik et al. 2021)	European Research on Management and Business Economics	Scopus
The Impact of University Innovation and Entrepreneurship Education on Entrepreneurial Intention From the Perspective of Educational Psychology	(Wang et al. 2021)	Frontiers in Psychology	Scopus
Visual Thinking Boosting Spanish Higher Education Students’ Entrepreneurial Intentions	(Gismera Tierno et al. 2021)	Journal of the Knowledge Economy	Scopus
Youth entrepreneurial intentions: an integrated model of individual and contextual factors	(Gulzar and Fayaz 2021)	International Journal of Organizational Analysis	Scopus
Determinants of entrepreneurial intentions of university students in selected post-communist countries in Europe: investigating cross-cultural differences	(Tomal and Szromnik 2021)	Journal of Business Economics and Management	ProQuest
Drivers of sustainable entrepreneurial intentions among university students: an integrated model from a developing world context	(Agu et al. 2021)	International Journal of Sustainability in Higher Education	Scopus
Motivational and Attitudinal Determinants of Entrepreneurial Intention: Hospitality and Tourism Students’ Perspectives	(Al-Jubari et al. 2021)	Journal of Hospitality and Tourism Education	Scopus
The moderating role of self-efficacy on the cognitive process of entrepreneurship: An empirical study in Vietnam	(Doanh 2021a)	International Journal of Entrepreneurship and Innovation Management	Scopus
Effects of Psychological and Cognitive Factors on the Relation between Entrepreneurial Intention and Academic Hazing: Case of the New Students in the Faculty of Social and Human Sciences at the University of Beira—Portugal	(Garcez and Franco 2021)	Entrepreneurship Research Journal	Scopus
An examination of the entrepreneurial intent of MBA students in Australia using the entrepreneurial intention questionnaire	(Lee-Ross 2017)	Journal of Management Development	ProQuest
The Impact of Self-efficacy on International Student Entrepreneur Intention	(Akadiri et al. 2017)	International Review of Management and Marketing	ProQuest
The influence of the dark triad on the relationship between entrepreneurial attitude orientation and entrepreneurial intention: A study among students in Taiwan University	(Do and Dadvari 2017)	Asia Pacific management review	Scopus
Entrepreneurial self-efficacy and intention among vietnamese students: a meta-analytic path analysis based on the theory of planned behavior	(Doanh and Bernat 2019)	Management Science Letters	Scopus
Determinants of individuals’ entrepreneurial intentions: a gender-comparative study	(Arshad et al. 2016)	Career Development International	Scopus
Determinants Influencing Entrepreneurial Intention among Undergraduates in Universities of Vietnam	(Bui et al. 2020)	Journal of Asian Finance, Economics and Business	Scopus
Entrepreneurial Intentions among University Students: The Moderating Role of Creativity	(Entrialgo and Iglesias 2020)	European Management Review	Scopus
Determinants of entrepreneurial intentions: Technical-vocational education and training students in Ethiopia	(Buli and Yesuf 2015)	Education and Training	Scopus
Investigating entrepreneurial intention among public sector university students of Pakistan	(Shah and Soomro 2017)	Education and Training	Scopus
Sports university education and entrepreneurial intentions: A comparison between Spain and Lithuania	(González-Serrano et al. 2018)	Education and Training	Scopus
Gender and university degree: a new analysis of entrepreneurial intention	(López-Delgado et al. 2019)	Education and Training	Scopus
Analyzing the Moderating Effect of Entrepreneurship Education on The Antecedents of Entrepreneurial Intention	(Bhat and Singh 2018)	Journal of entrepreneurship education	Scopus
How does the public and private university environment affect students’ entrepreneurial intention?	(Canever et al. 2017)	Education and Training	Scopus
Entrepreneurial intention of international business students in Viet Nam: a survey of the country joining the Trans-Pacific Partnership	(Nguyen 2017)	Journal of Innovation and Entrepreneurship	Scopus
Entrepreneurial Intentions of Agricultural Students: Levels and Determinants	(Pouratashi 2015)	Journal of Agricultural Education and Extension	Scopus
Tool for measuring the influence of the field of knowledge on entrepreneurial intention among university students	(Díez-Echavarría et al. 2020)	Periodica Polytechnica Social and Management Sciences	Scopus
Entrepreneurial intentions of university students: An international comparison between African, European and Canadian students	(St-Jean et al. 2014)	International Journal of Entrepreneurship and Innovation Management	Scopus
Short-Term and Long-Term Entrepreneurial Intention Comparison between Pakistan and Vietnam	(Nasar et al. 2019)	Sustainability (Switzerland)	Scopus
The impact of entrepreneurial alertness on entrepreneurial intention among university students in Malaysia: Theory of planned behaviour	(Lim et al. 2017)	Journal of Engineering and Applied Sciences	Scopus
Determinants of entrepreneurial intention among engineering students based on structural equation modeling	(Arango-Botero et al. 2020)	Pertanika Journal of Social Sciences and Humanities	Scopus
Determinants of personal attitudes dimensions on entrepreneurial intentions	(Ashokan et al. 2019)	International Journal of Engineering and Advanced Technology	Scopus
Determinants of students' entrepreneurial intention—a comparative study in Poland and Hong Kong	(Law and Jakubiak 2020)	International Journal of Innovation and Learning	Scopus
Family education? Unpacking parental factors for tourism and hospitality students’ entrepreneurial intention	(Liu and Zhao 2021)	Journal of Hospitality, Leisure, Sport and Tourism Education	Scopus
Factors affecting entrepreneurial intention among tourism undergraduate students in Vietnam	(Phuc et al. 2020)	Management science letters	Scopus
Academic, family, and peer influence on entrepreneurial intention of engineering students	(Lingappa et al. 2020)	SAGE Open	Scopus
Determinants of social entrepreneurial intentions for educational programs	(Asma et al. 2019)	Journal of Public Affairs	Scopus
The validity and reliability of thriving scale in academic context: Mindfulness, GPA, and entrepreneurial intention among university students	(Ozcan et al. 2021)	Current Psychology	Scopus
Personal factors, entrepreneurial intention, and entrepreneurial status: A multinational study in three institutional environments	(Schlaegel et al. 2021)	Journal of International Entrepreneurship	Scopus
An analysis of the determinants of entrepreneurial intentions among students: A Romanian case study	(Popescu et al. 2016)	Sustainability (Switzerland)	Scopus
Psychological characteristics and entrepreneurial intention: A study among university students in North Borneo, Malaysia	(Nasip et al. 2017)	Education and Training	Scopus
What Makes a Media Entrepreneur? Factors Influencing Entrepreneurial Intention of Mass Communication Students	(Buschow and Laugemann 2020)	Journalism and Mass Communication Educator	Scopus
Entrepreneurial characteristics amongst university students: insights for understanding entrepreneurial intentions amongst youths in a developing economy	(Ibidunni et al. 2021)	Education and Training	Scopus
Impact of personality traits on entrepreneurial intention and demographic factors as moderator	(Yasir et al. 2019)	International Journal of Entrepreneurship	Scopus
South African university students' entrepreneurial intention as a correlate of entrepreneurship risk perceptions and aversion	(Mahola et al. 2019)	Journal of Human Ecology	Scopus
The Interplay between the Psychological Factors and Entrepreneurial Intention: An Empirical Investigation	(Shahneaz et al. 2020)	Journal of Asian Finance, Economics and Business	Scopus
Factors associated with entrepreneurial intentions in doctor of pharmacy students	(Huston 2018)	American Journal of Pharmaceutical Education	Scopus
Psychological Capital and University Students’ Entrepreneurial Intention in China: Mediation Effect of Entrepreneurial Capitals	(Zhao et al. 2020)	Frontiers in Psychology	Scopus
A non-parametric analysis of the relationship between business experience and entrepreneurial intention of final-year university students	(Hudea et al. 2021)	Mathematics	Scopus
An empirical study on entrepreneurial intentions among Japanese university students	(Fukuda 2014)	International Journal of Entrepreneurship and Small Business	Scopus
Linking entrepreneurship education and entrepreneurial intentions: An interactive effect of social and personal factors	(Shahid and Ahsen 2021)	International Journal of Learning and Change	Scopus
Context, gender and entrepreneurial intentions: How entrepreneurship education changes the equation	(van Ewijk and Belghiti-Mahut 2019)	International Journal of Gender and Entrepreneurship	Scopus
Can the entrepreneurship course improve the entrepreneurial intentions of students?	(Chen et al. 2015)	International entrepreneurship and management journal	Scopus
Teachers as entrepreneurial role models: The impact of a teacher's entrepreneurial experience and student learning styles in entrepreneurial intentions	(Diegoli et al. 2018)	Journal of entrepreneurship education	Scopus
Boosting entrepreneurial intention of university students: Is a serious business game the key?	(Pérez-Pérez et al. 2021)	International Journal of Management Education	Scopus
Personal values as predictors of entrepreneurial intentions of university students	(Sánchez 2021)	Journal of Evolutionary Studies in Business	Scopus
The effect of intrinsic and extrinsic factors on entrepreneurial intentions: The moderating role of collectivist orientation	(Arshad et al. 2019)	Management Decision	Scopus
Sustainability orientation and sustainable entrepreneurial intentions of University students in South Africa	(Fatoki 2019)	Entrepreneurship and Sustainability Issues	Scopus
An assessment of entrepreneurial intention among university students in Cameroon	(Neneh 2014)	Mediterranean Journal of Social Sciences	Scopus
Can sense of opportunity identification efficacy play a mediating role? Relationship between network embeddedness and social entrepreneurial intention of university students	(Wang et al. 2019)	Frontiers in Psychology	Scopus
Assessing the role of socio-economic values on entrepreneurial intentions among university students in Cape Town	(Kalitanyi and Bbenkele 2017)	South African Journal of Economic and Management Sciences	Scopus
Cultural values as determinants of entrepreneurial intentions among university students in Cape Town-South Africa	(Kalitanyi and Bbenkele 2018)	Journal of Enterprising Communities	Scopus
Personality Traits, Demographic Factors and Entrepreneurial Intentions: Improved Understanding from a Moderated Mediation Study	(Ahmed et al. 2021)	Entrepreneurship Research Journal	Scopus
Attitude and Alertness in Personality Traits: A Pathway to Building Entrepreneurial Intentions Among University Students	(Biswas and Verma 2021)	Journal of Entrepreneurship	Scopus
Undergraduates entrepreneurial intention: Holistic determinants matter	(Kowang et al. 2021)	International Journal of Evaluation and Research in Education	Scopus
The Roles of Psychological Capital and Gender in University Students’ Entrepreneurial Intentions	(Margaça et al. 2021)	Frontiers in Psychology	Scopus
The students’ attitudes and entrepreneurial intention: Evidence from Vietnam universities	(Phuong et al. 2021)	Management science letters	Google Scholar
Determinants Of Entrepreneurial Intention: Empirical Study Of Student Entrepreneurs	(Rakib et al. 2020)	Academy of Entrepreneurship Journal	Scopus
Predicting entrepreneurial intention across the university	(Bell 2019)	Education and Training	Scopus
Entrepreneurial Potential and Gender Effects: The Role of Personality Traits in University Students’ Entrepreneurial Intentions	(Ward et al. 2019)	Frontiers in Psychology	Scopus
Determinants of entrepreneurial intentions: an international cross-border study	(Fernandes et al. 2018)	International Journal of Innovation Science	Scopus
Modelling green entrepreneurial intention among university students using the entrepreneurial event and cultural values theory	(Ramayah et al. 2019)	International Journal of Entrepreneurial Venturing	Scopus
Exploring Factors Surrounding Students’ Entrepreneurial Intentions in Medical Informatics: The Theory of Planning Behavior Perspective	(Wu et al. 2020)	Frontiers in Psychology	Scopus
Exploring factors motivating entrepreneurial intentions: the case of Italian university students	(Ferri et al. 2019)	International Journal of Training and Development	Scopus
Entrepreneurial Intentions of University Students: A Study of Design Undergraduates in Spain	(Ubierna et al. 2014)	Industry and Higher Education	Scopus
Relationship between psychological factors and entrepreneurial intentions of university undergraduates in north east, Nigeria	(Bello and Danjuma 2017)	Journal of Technical Education and Training	Scopus
Investigating the relationship between educational support and entrepreneurial intention in Vietnam: The mediating role of entrepreneurial self-efficacy in the theory of planned behavior	(Maheshwari and Kha 2021)	The International Journal of Management Education	Scopus
The role of contextual factors on predicting entrepreneurial intention among Vietnamese students	(Doanh 2021b)	Entrepreneurial Business and Economics Review	Scopus
Entrepreneurial intention among female university students in Oman	(Echchabi et al. 2020)	Journal for International Business and Entrepreneurship Development	Scopus
The effects of internal and external barriers on Vietnamese students’ entrepreneurial intention	(Thanh et al. 2020)	Management science letters	Scopus
Entrepreneurial intention of Bangladeshi students: Impact of individual and contextual factors	(Hossain et al. 2019)	Problems and Perspectives in Management	Scopus
Factors influencing social entrepreneurial intentions of students at a university in South Africa	(Chinaire et al. 2021)	Academy of Entrepreneurship Journal	Scopus
Entrepreneurial intentions of Colombian business students: Planned behaviour, leadership skills and social capital	(Henley et al. 2017)	International journal of entrepreneurial behaviour & research	Scopus
What factors affect the entrepreneurial intention to start-ups? The role of entrepreneurial skills, propensity to take risks, and innovativeness in open business models	(Shahzad et al. 2021)	Journal of Open Innovation: Technology, Market, and Complexity	Scopus
Pathways toward entrepreneurial intention among Malaysian universities’ students	(Bazkiaei et al. 2021)	Business Process Management Journal	Scopus
Factors influencing entrepreneurial intentions the most for university students in Vietnam: educational support, personality traits or TPB components?	(Maheshwari 2021)	Education and Training	Scopus
Influence of educational programs oriented toward entrepreneurship on the entrepreneurial intention of university students: the case of Chile [La influencia de programas de aprendizaje orientados al emprendimiento en la intención emprendedora de estudiantes universitarios: el caso de Chile]	(Silva et al. 2021)	Academia Revista Latinoamericana de Administracion	Scopus
Do entrepreneurial education and big-five personality traits predict entrepreneurial intention among universities students?	(Bazkiaei et al. 2020)	Cogent Business & Management	Scopus
Relationship between entrepreneurship education and innovative start-up intentions among university students	(Hien and Cho 2018)	International journal of entrepreneurship	Scopus
Determinants of students’ entrepreneurial intention: An empirical research [Empirijsko istraživanje odrednica studentske poduzetničke namjere]	(Zovko et al. 2020)	Management (Croatia)	Scopus
Integrating and extending competing intention models to understand the entrepreneurial intention of senior university students	(Eid et al. 2019)	Education and Training	Scopus
Factors impacting entrepreneurial intentions among university students in Saudi Arabia: testing an integrated model of TPB and EO	(Al-Mamary et al. 2020)	Education and Training	Scopus
University entrepreneurial push strategy and students’ entrepreneurial intention	(Wegner et al. 2020)	International journal of entrepreneurial behaviour & research	Scopus
An investigation of factors influencing entrepreneurial intention amongst university students	(Kör et al. 2020)	Journal of Higher Education Theory and Practice	Scopus
Exploring the link between entrepreneurship education and entrepreneurial intentions: the moderating role of educational fields	(Duong 2021)	Education and Training	Scopus
The impact of individual and environmental characteristics on students’ entrepreneurial intention	(Duong et al. 2020)	Management science letters	Scopus
Influence of the link between resources and behavioural factors on the entrepreneurial intentions of electrical installation and maintenance work students	(Ohanu and Shodipe 2021)	Journal of Innovation and Entrepreneurship	Scopus
Social entrepreneurial intentions and its influential factors: A comparison of students in Taiwan and Hong Kong	(Hsu and Wang 2019)	Innovations in Education and Teaching International	Scopus
The role of structural support in predicting entrepreneurial intention: Insights from Vietnam	(Van Trang and Doanh 2019)	Management science letters	Scopus
The Impact of Access to Finance and Environmental Factors on Entrepreneurial Intention: The Mediator Role of Entrepreneurial Behavioural Control	(Nguyen 2020)	Entrepreneurial Business and Economics Review	Scopus
A conceptual study on factors of entrepreneurial potentiality and their impact on entrepreneurial intention with the moderating role of entrepreneurship education	(Aggarwal 2019)	Prabandhan: Indian Journal of Management	Scopus
Perceived employability and entrepreneurial intentions across university students and job seekers in Togo: The effect of career adaptability and self-efficacy	(Atitsogbe et al. 2019)	Frontiers in Psychology	Scopus
Entrepreneurial intention of engineering students and associated influence of contextual factors/Intención emprendedora de los estudiantes de ingeniería e influencia de factores contextuales	(Morales-Alonso et al. 2016)	Revista de Psicologia Social	Scopus
Factors influencing entrepreneurial intentions of undergraduate agricultural students in Nigeria [Nijerya’da ziraat fakültesi öğrencilerinin girişimciliklerini etkileyen faktörler]	(Adeyonu et al. 2019)	Yuzuncu Yil University Journal of Agricultural Sciences	Scopus
The impact of network ties, shared languages and shared visions on entrepreneurial intentions of online university students	(Pérez-Macías et al. 2020)	Studies in Higher Education	Scopus
The effect of psychological and contextual factors on the entrepreneurial intention of university students in South Africa	(Dzomonda et al. 2015)	Corporate Ownership and Control	Scopus
An exploratory study of entrepreneurial intention among university students in Ghana	(Amanamah et al. 2018)	International Journal of Scientific and Technology Research	Scopus
Gender and Social Legitimacy of Entrepreneurship: Contribution to Entrepreneurial Intention in University Students from Chile and Colombia	(Soria et al. 2016)	Journal of technology management & innovation	Scopus
Factors influencing e-entrepreneurial intention among female students in Saudi Arabia	(Alzamel et al. 2020)	International Journal of Criminology and Sociology	Scopus
The influence of gender on the entrepreneurial intentions of journalism students	(Caro-González et al. 2017)	Intangible Capital	Scopus
The influence of culture on entrepreneurial intentions: a Nigerian university graduates' perspective	(Chukwuma-Nwuba 2018)	Transnational Corporations Review	Scopus
Entrepreneurial intention among online and face-to-face university students: The influence of structural and cognitive social capital dimensions	(Pérez-Macías et al. 2021)	Journal of International Entrepreneurship	Scopus
Entrepreneurial intentions of university students: a case-based study	(Palalić et al. 2017)	Journal of Enterprising Communities	Scopus
A psychosocial study of self-perceived creativity and entrepreneurial intentions in a sample of university students	(Laguía et al. 2019)	Thinking Skills and Creativity	Scopus
Model of the entrepreneurial intention of university students in the Pearl River Delta of China	(Hou et al. 2019)	Frontiers in Psychology	Scopus
Determinants of university students' entrepreneurial intention: GUESSS Colombia study	(Cano and Tabares 2017)	Espacios	Scopus
Exploring the factors responsible in predicting entrepreneurial intention among nascent entrepreneurs: A field research	(Tiwari et al. 2020)	South Asian Journal of Business Studies	Scopus
Relationship among influential factors of entrepreneurial intention in terms of gender: Case of postgraduate students in Malaysia	(Yaghmaei et al. 2015)	Mediterranean Journal of Social Sciences	Scopus
Validating the Entrepreneurial Intention Model on the University Students in Saudi Arabia	(Hoda et al. 2020)	Journal of Asian Finance, Economics and Business	Scopus
Entrepreneurial intentions of private university students in the kingdom of Bahrain	(Al-Shammari and Waleed 2018)	International Journal of Innovation Science	Scopus
Entrepreneurial intention among Latin American university students [Intención emprendedora entre estudiantes universitarios en Latinoamérica]	(Leiva et al. 2021)	Academia Revista Latinoamericana de Administracion	Scopus
Comparing the Entrepreneurial Intention between Female and Male Engineering Students	(Lo et al. 2017)	Journal of Women's Entrepreneurship and Education (JWEE)	Google Scholar
How University Entrepreneurship Support Affects College Students’ Entrepreneurial Intentions: An Empirical Analysis from China	(Lu et al. 2021)	Sustainability (Switzerland)	Scopus
Factors influencing entrepreneurial intention of university students in china: Integrating the perceived university support and theory of planned behavior	(Su et al. 2021)	Sustainability (Switzerland)	Scopus
A structural model for predicting entrepreneurial intention of university students	(Tassawa 2019)	Humanities, Arts and Social Sciences Studies	Scopus
Fostering sustainable businesses: understanding sustainability-driven entrepreneurial intention among university students in Pakistan	(Waris et al. 2021)	Social Responsibility Journal	Scopus
A Model of Factors Affecting Entrepreneurial Intention among Information Technology Students in Vietnam	(Vuong et al. 2020)	Journal of Asian Finance, Economics and Business	Scopus
Entrepreneurial intention among female university students: Examining the moderating role of entrepreneurial education	(Anwar et al. 2020)	Journal for International Business and Entrepreneurship Development	Scopus
Determinants of entrepreneurial intention among undergraduates in a Muslim community	(Ezeh et al. 2020)	Management Research Review	Scopus
Entrepreneurial Intention: Creativity, Entrepreneurship, and University Support	(Anjum et al. 2021a)	Journal of open innovation	Scopus
Effect of memorial university's environment & support system in shaping entrepreneurial intention of students	(Bazan et al. 2019)	Journal of Entrepreneurship Education	Scopus
Entrepreneurship education and entrepreneurial intentions of university students in Vietnam: the mediating roles of self-efficacy and learning orientation	(Hoang et al. 2021)	Education and Training	Scopus
The impact of entrepreneurial education on entrepreneurial intention: The case of Vietnamese	(Doan and Phan 2020)	Management science letters	Scopus
Influence of university-related factors on students’ entrepreneurial intentions	(Lopez and Alvarez 2019)	International Journal of Entrepreneurial Venturing	Scopus
The relationship between higher education and entrepreneurial intention among Vietnamese students	(Kim et al. 2020)	Management science letters	Scopus
The analysis of the effect of entrepreneurship education, perceived desirability, and entrepreneurial self-efficacy on university students' entrepreneurial intention	(Suratno and Kusmana 2019)	Universal Journal of Educational Research	Scopus
Factors affecting the entrepreneurial intentions among university students of Thailand	(Tamprateep et al. 2019)	Journal of Computational and Theoretical Nanoscience	Scopus
Role of entrepreneurial education in shaping entrepreneurial intention among university students: Testing the hypotheses using mediation and moderation approach	(Anwar et al. 2022)	Journal of Education for Business	Scopus
Perceived university support, entrepreneurial self-efficacy, heterogeneous entrepreneurial intentions in entrepreneurship education: The moderating role of the Chinese sense of face	(Shi et al. 2020)	Journal of Entrepreneurship in Emerging Economies	Scopus
Characterization of entrepreneurial intention in university students as from Systemic Entrepreneurship Intention Model: A case study	(Velásquez et al. 2018)	Cuadernos de Gestion	Scopus
Entrepreneurial intentions of university students in Colombia: Exploration based on the theory of planned behavior	(Rueda Barrios et al. 2022)	Journal of Education for Business	Scopus
University entrepreneurial intentions: mainland and insular regions – are they different?	(Lopes et al. 2020)	Education and Training	Scopus
Entrepreneurial intention and obstacles of undergraduate students: the case of the universities of Andalusia	(Arranz et al. 2019)	Studies in Higher Education	Scopus
Entrepreneurial intention of Indian university students: the role of opportunity recognition and entrepreneurship education	(Hassan et al. 2020)	Education and Training	Scopus
Entrepreneurial intention models as applied to Latin America	(Guzmán-Alfonso and Guzmán-Cuevas 2012)	Journal of Organizational Change Management	Scopus
The role of autonomy as a predictor of entrepreneurial intention among university students in Yemen	(Al-Jubari et al. 2017)	International Journal of Entrepreneurship and Small Business	Scopus
The Role of Personal Values in Forming Students' Entrepreneurial Intentions in Developing Countries	(Karimi and Makreet 2020)	Frontiers in Psychology	Scopus
University Students’ Behaviour towards Entrepreneurial Intention in Ecuador: Testing for the Influence of Gender	(Rodriguez-Gutierrez et al. 2020)	International Journal of Environmental Research and Public Health	Scopus
Contextual Motivations for Undergraduates’ Entrepreneurial Intentions in Emerging Asian Economies	(Looi 2020)	The Journal of Entrepreneurship	Scopus
Entrepreneurial Intention: A Gender Study in Business and Economics Students from Chile	(Contreras-Barraza et al. 2021)	Sustainability (Switzerland)	Scopus
Impact of behavioural factors as related to available resources on entrepreneurial intentions of electrical installation and maintenance works students	(Benedictohanu et al. 2020)	International Journal of Engineering Education	Scopus
Factors Affecting the Entrepreneurial Intention of Students Pursuing Journalism and Media Studies: Evidence from Spain	(Goyanes 2015)	JMM International Journal on Media Management	Scopus
Linking entrepreneurial intentions and mindset models: A comparative study of public and private universities in Vietnam	(Cao and Ngo 2019)	Gadjah Mada International Journal of Business	Scopus
Social and economic factors affecting the entrepreneurial intention of university students	(Belas et al. 2017)	Transformations in Business and Economics	Scopus
Determinants of entrepreneurial intention among Sudanese university students	(Ali and Abou 2020)	Management science letters	Scopus
Determinants of entrepreneurial intention of pharmacy students in Chennai	(Sankar and Irin Sudha 2016)	International Journal of Pharmaceutical Sciences Review and Research	Scopus
Do Islamic Values Impact Social Entrepreneurial Intention of University Students in Malaysia? An Empirical Investigation Into The Mediating Role of Empathy	(Mohammadi et al. 2020)	International Journal of Economics and Management	Scopus
Entrepreneurial intentions—theory and evidence from Asia, America, and Europe	(Paul et al. 2017)	Journal of International Entrepreneurship	Scopus
Exploring entrepreneurial intentions in Latin American university students	(Torres et al. 2017)	International Journal of Psychological Research	Scopus
The role of social and psychological factors on entrepreneurial intention among islamic college students in Indonesia	(Ahamed and Rokhman 2015)	Entrepreneurial Business and Economics Review	Scopus
Research on the influencing factors of entrepreneurial intentions based on mediating effect of self-actualization	(Dong et al. 2019)	International Journal of Innovation Science	Scopus
Do Entrepreneurship Students Have an Intention to Become an Entrepreneur?	(Kurniawan et al. 2019)	Journal of entrepreneurship education	Scopus
Planned behavior and religious beliefs as antecedents to entrepreneurial intention: A study with university students [Comportamento planejado e crenças religiosas como antecedentes da intenção empreendedora: Um estudo com universitários]	(Paiva et al. 2020)	Revista de Administracao Mackenzie	Scopus
Factors influencing the entrepreneurial intention of business graduate students of Saudi Arabia University	(Gangwani and Ballout 2020)	Academy of Entrepreneurship journal	Scopus
Determinants of students’ entrepreneurial intention to compete in a fast-pitch competition	(Jang et al. 2019)	Journal of Education for Business	Scopus
Determinants of entrepreneurial intention among generation y students within the Johannesburg Metropolitan Area of South Africa	(Maziriri et al. 2019)	African Journal of Business and Economic Research	Scopus
Assessing the university students’ entrepreneurial intention: Entrepreneurial education and creativity	(Saptono et al. 2019)	Humanities and Social Sciences Reviews	Scopus
Entrepreneurial intentions among university students: A case study of Durban University of Technology	(Jwara and Hoque 2018)	Academy of Entrepreneurship journal	Scopus
A critical comparison of factors affecting science and technology students’ entrepreneurial intention: a tale of two genders	(Roy and Das 2020)	International Journal for Educational and Vocational Guidance	Scopus
The mediating role of entrepreneurial passion in the relationship between entrepreneur education and entrepreneurial intention among university students in Thailand	(Sriyakul and Jermsittiparsert 2019)	International Journal of Innovation, Creativity and Change	Scopus
Determinants of entrepreneurial intention: Empirical insights from Malaysian undergraduate business students	(Ali et al. 2017)	International Journal of Economic Research	Scopus
Entrepreneurial education and entrepreneurial intention: The mediating role of creativity disposition among University Students in Thailand	(Rungsrisawat and Sutduean 2019)	International Journal of Innovation, Creativity and Change	Scopus
Effects of entrepreneurial knowledge on entrepreneurial intentions: a longitudinal study of selected South-east Asian business students	(Roxas 2014)	Journal of Education and Work	Scopus
Factors influencing entrepreneurial intention: A case of students in a South African University	(Nsahlai et al. 2020)	Academy of Entrepreneurship journal	Scopus
Determinants of entrepreneurial intentions: An institutional embeddedness perspective	(Shahid et al. 2018)	Journal of Small Business and Entrepreneurship	Scopus
Psycho-demographic Factors and Entrepreneurial Intention Among University Students	(Ojewumi et al. 2020)	Open Journal for Psychological Research	Google Scholar
Factors affecting entrepreneurial intention among UniSZA students	(Ghazali et al. 2012)	Asian Social Science	Scopus
Effect of demographic factors on entrepreneurial intention of management students in Nagpur university, India	(Uike 2019)	International Journal of Scientific and Technology Research	Scopus
What impact entrepreneurial intention? Cultural, environmental, and educational factors	(Ao and Liu 2014)	Journal of Management Analytics	Scopus
Understanding the entrepreneurial intention in the light of contextual factors: Gender analysis	(Rahaman et al. 2020)	Journal of Asian Finance, Economics and Business	Scopus
Symmetric and asymmetric modeling of entrepreneurial ecosystem in developing entrepreneurial intentions among female university students in Saudi Arabia	(Ali et al. 2019)	International journal of gender and entrepreneurship	Scopus
Students' Attitudes toward Entrepreneurship at the Arab Open University-Lebanon	(Hendieh et al. 2019)	Journal of entrepreneurship education	Scopus
Determinants of entrepreneurial intention among millennial generation in emerging countries	(Wibowo et al. 2019)	Journal of Legal, Ethical and Regulatory Issues	Scopus
Determinants of eco entrepreneurial intention among students: Study in the entrepreneurial education practices	(Nuringsih and Puspitowati 2017)	Advanced Science Letters	Scopus
Factors affecting entrepreneurial intention of Senior University Students	(Duque Aldaz et al. 2018)	Espacios	Scopus
Indonesian university students' entrepreneurial intention: A conceptual study	(Pradana et al. 2020)	Journal of Critical Reviews	Scopus
A comparative analysis of entrepreneurial intention and migration attitudes of students in Vietnam and Poland	(Le Trung et al. 2020)	Management science letters	Scopus
Social values as determinants of entrepreneurial intentions among university students in Cape Town—South Africa	(Kalitanyi and Visser 2016)	Problems and perspectives in management	Scopus
Sociological and theory of planned behaviour approach to understanding entrepreneurship: Comparison of Vietnam and South Korea	(Nguyen et al. 2020)	Cogent business & management	Scopus
Determinants of Entrepreneurial Intentions Among Romanian Students	(Popescu et al. 2016)	Transformations in Business and Economics	Scopus
How does the perception of entrepreneurial ecosystem affect entrepreneurial intention among university students in Saudi Arabia?	(Elnadi et al. 2020)	International Journal of Entrepreneurship	Scopus
Factors influencing entrepreneurial intentions among arab students	(Al Bakri and Mehrez 2017)	International Journal of Entrepreneurship	Scopus
Relationships between students' work values and entrepreneurial intention among Vietnamese students	(Van Trang et al. 2020)	Academy of Entrepreneurship journal	Scopus
Corruption shock in Mexico: FsQCA analysis of entrepreneurial intention in university students	(Castelló-Sirvent and Pinazo-Dallenbach 2021)	Mathematics	Scopus
Influence of institutional environment on entrepreneurial intention: a comparative study of two countries university students	(Díaz-Casero et al. 2012)	International Entrepreneurship and Management Journal	Scopus
Parental and gender effects on the entrepreneurial intention of university students in South Africa	(Fatoki 2014)	Mediterranean Journal of Social Sciences	Scopus
Influence of demographic factors on the entrepreneurial intentions of university students in Oman	(Uddin et al. 2016)	Investment Management and Financial Innovations	Scopus
The effect of educational background on entrepreneurial intention	(Xuan et al. 2020)	Management science letters	Scopus
A decade of entrepreneurship education in the Asia Pacific for future directions in theory and practice	(Wu and Wu 2017)	Management decision	Scopus
Contextual factors and their effects on future entrepreneurs in China: A comparative study of entrepreneurial intentions	(Bernhofer and Han 2014)	International Journal of Technology Management	Scopus
Factors influencing entrepreneurial intention and the moderating role of entrepreneurship education: A conceptual model	(Shamsudin et al. 2017)	Advanced Science Letters	Scopus
University Students' Entrepreneurial Intentions: A Comparative Analysis of Hong Kong and Guangzhou	(Bickenbach et al. 2017)	China and World Economy	Scopus
Determinants of entrepreneurial intention among university business students in Kenya: Lessons from Kenyatta University	(Kilonzo and Nyambegera 2014)	International Journal of Entrepreneurship and Small Business	Scopus
Tracking the cyber entrepreneurial intention of private universities students in Malaysia	(Ismail et al. 2012)	International Journal of Entrepreneurship and Small Business	Scopus
Descriptive research study on factors influencing entrepreneurial intention among engineering students in Virudhunagar District	(Sumathi et al. 2018)	Journal of Advanced Research in Dynamical and Control Systems	Scopus
An examination of university student entrepreneurial intentions by type of venture	(Carey et al. 2010)	Journal of Developmental Entrepreneurship	Scopus
Impact of academic majors on entrepreneurial intentions of Vietnamese students: An extension of the theory of planned behavior	(Dao et al. 2021)	Heliyon	Scopus
